# An In Vitro Analysis of TKI-Based Sequence Therapy in Renal Cell Carcinoma Cell Lines

**DOI:** 10.3390/ijms24065648

**Published:** 2023-03-15

**Authors:** Angela Zaccagnino, Bozhena Vynnytska-Myronovska, Michael Stöckle, Kerstin Junker

**Affiliations:** Department of Urology and Pediatric Urology, Saarland University, 66421 Homburg, Germanymichael.stoeckle@uks.eu (M.S.); kerstin.junker@uks.eu (K.J.)

**Keywords:** renal cell carcinoma, sunitinib resistance, tyrosine kinase inhibitor, sequential treatment, receptor tyrosine kinases, drug resistance, cell signaling

## Abstract

The tyrosine kinase inhibitor (TKI) cabozantinib might impede the growth of the sunitinib-resistant cell lines by targeting MET and AXL overexpression in metastatic renal cell carcinoma (mRCC). We studied the role of MET and AXL in the response to cabozantinib, particularly following long-term administration with sunitinib. Two sunitinib-resistant cell lines, 786-O/S and Caki-2/S, and the matching 786-O/WT and Caki-2/WT cells were exposed to cabozantinib. The drug response was cell-line-specific. The 786-O/S cells were less growth-inhibited by cabozantinib than 786-O/WT cells (*p*-value = 0.02). In 786-O/S cells, the high level of phosphorylation of MET and AXL was not affected by cabozantinib. Despite cabozantinib hampering the high constitutive phosphorylation of MET, the Caki-2 cells showed low sensitivity to cabozantinib, and this was independent of sunitinib pretreatment. In both sunitinib-resistant cell lines, cabozantinib increased Src-FAK activation and impeded mTOR expression. The modulation of ERK and AKT was cell-line-specific, mirroring the heterogeneity among the patients. Overall, the MET- and AXL-driven status did not affect cell responsiveness to cabozantinib in the second-line treatment. The activation of Src-FAK might counteract cabozantinib activity and contribute to tumor survival and may be considered an early indicator of therapy response.

## 1. Introduction

Renal cell carcinoma (RCC) is the seventh most common form of neoplasm in developed countries, accounting for approximately 3% of all diagnosed cancers worldwide [1,2], with an annual increase in incidence of approximately 2%, leading to approximately 400,000 new cases diagnosed and approximately 180,000 kidney-cancer-related deaths [3,4,5].

At present, therapeutic approaches used to treat patients with metastatic RCC (mRCC) are based on specific molecular characteristics that play an essential role in disease development [6]. In clear cell RCC (ccRCC, 80% of cases), the mutation of the tumor-suppressor von Hippel–Lindau (*VHL*) gene; chromosome remodeling by *PBRM1*, *BAP1* and *SETD2* genes; hyperactivated PI3K/AKT pathways; and mTOR alterations are the main molecular features [7,8,9,10]. A loss of *VHL* elevates the stability of the hypoxia-inducible factor (HIF) transcription factor, which, in turn, tunes the expression of hypoxia-responsive genes, vascular endothelial growth factor (VEGF), platelet-derived growth factor beta (PDGF-β) and transforming growth factor (TGF-β) associated with both aberrant angiogenesis and tumor growth [11,12,13,14]. The small molecule receptor tyrosine kinase inhibitors (TKIs) sunitinib, pazopanib, sorafenib and cabozantinib can primarily hamper VEGFR and PDGFR receptors by preventing the phosphorylation of tyrosine residues on the catalytic receptors [15,16]. This, in turn, blocks their ability to convey their signals to cellular replicative machinery, promoting angiogenesis, metastasis and protection from apoptosis [17,18]. Systemic therapy has recently significantly evolved with the use of immune checkpoint inhibitors (ICIs) [19], which restore T-cell-mediated antitumor immune responses [20]. Although the combination of ICI and TKI (IC–IC and IC–TKI) has improved patient outcomes [21,22] and is considered the front-line standard of care [23] for RCC treatment, TKI monotherapy remains a treatment option for selected patient profiles (i.e., patients with a compromised immune system who are not suitable candidates for ICI therapy) [23,24]. However, the development of acquired resistance is a frequent problem that limits the clinical use of TKIs despite an initially strong antitumor effect. Multiple studies have described acquired resistance to sunitinib in tumor cells [25] as a transient form of insensitivity to VEGFR inhibition [26] that could likely be driven by dynamic epigenetic regulation [27,28,29] rather than a stable genetic form of resistance. Therefore, VEGFR-TKI rechallenge with dose escalation or switching to another established TKI is considered an option in the second-line setting, as well as consideration of different drug-specific target profiles [15]. In this regard, sunitinib resistance can induce compensatory overexpression/activation of the receptor tyrosine kinases MET and AXL, as well as their transduction signaling in tumor cells. In addition, a loss of *VHL*, along with hypoxia, can promote MET expression, which has also been associated with worse outcomes in RCC [30,31,32,33]. Cabozantinib targeting of multireceptor tyrosine kinases MET, AXL and VEGFR is recommended at present as a first-line therapeutic treatment option in intermediate and unfavorable risk groups [34], as well as second-line therapy for patients with metastatic RCCs [35]. Whereas clinical data have shown improved progression-free survival (PFS) and overall survival (OS) rates [36,37], little information is available on the biological effects of cabozantinib alone or in sequence following sunitinib therapy. The limited number of studies on TKI-based sequence treatment suggests that the overexpression of receptor tyrosine kinases (RTKs) following first-line treatment may increase the sensitivity of tumor cells to cabozantinib in second-line treatment [12,30,38]. To date, few biomarkers of response to therapy have been identified, with inadequate understanding of the biological and molecular bases for selecting cabozantinib as a second-line treatment following long-term exposure to sunitinib. Moreover, little is known about the complex molecular heterogeneity among RCC patients, which is based on genetic, epigenetic and molecular pathways [39] and which might, in turn, influence individual drug sensitivities across patients.

In the present study, we investigated the role and possible mechanism of MET and AXL activation in response to cabozantinib, particularly following the long-term administration of sunitinib to patients. Preclinical testing seldom considers second treatment with a TKI in tumor cell models that have experienced long-term exposure to first-line TKI, closely resembling the clinical setting. Therefore, we used two age-matched cell lines, i.e., the treatment-naïve (wild type) and corresponding sunitinib-treated cell lines, which were challenged with a single dose of sunitinib (3 µM) over four months (long-term treatment). We analyzed the effects of cabozantinib both alone and in sequence on cell survival and on the modulation of signaling transduction proteins that are responsible for cell proliferation, survival, and migration. This provides a better understanding of the molecular pathways related to drug responses that can be integrated with the data obtained from large clinical trials to improve the selection of patients for TKI-based sequence treatment.

## 2. Results

### 2.1. The Cytotoxic Effect of Cabozantinib on Sunitinib-Treated and Treatment-Naïve Human RCC Cell Lines Is Cell-Line-Specific

**Figure 1:**
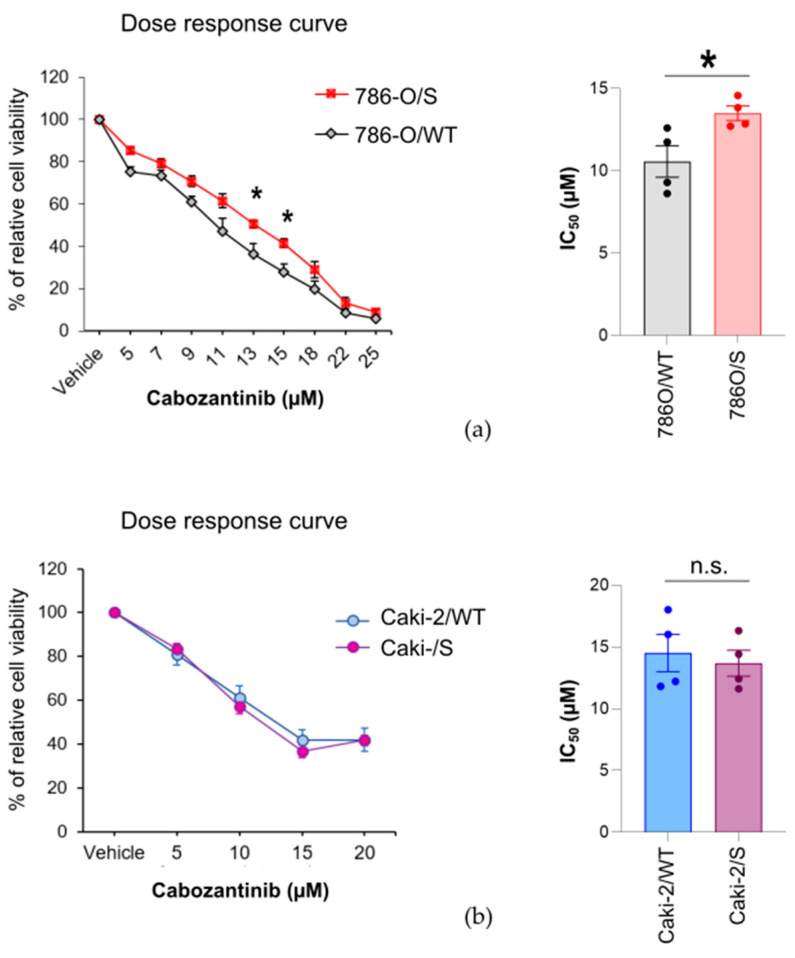
Cytotoxic effect of cabozantinib on human RCC treatment-naïve (wild type) and sunitinib-treated cell lines. Following chronic exposure to sunitinib for 4 months, 786-O/S and Caki-2/S cell lines were subjected to drug withdrawal for 5 days. The paired 786-O/WT and 786-O/S (**a**) and Caki-2/WT and Caki-2/S (**b**) cell lines were treated with increasing concentrations of cabozantinib for 72 h. The number of viable cells was assessed by the WST-1 test. The data are expressed as averages ± SE from independent experiments (n = 4). (***a***) A dose–response curve (**left**) was generated, and the IC_50_ values of cabozantinib were compared between the 786-O/WT and 786-O/S cells (**right**). (***b***) Caki-2 cell lines showed an incomplete dose–response curve (**left**), and a negative control value (DMSO) and a positive control cell line (786-O) for a maximally inhibited response were used to constrain the curve and obtain the IC_50_ value (**right**). Asterisks (*) indicate significant differences between sunitinib-resistant and wild-type cell lines (*p* < 0.05). The figure was generated with Inkscape (version 1.0.2, RRID: SCR_014479).

We aimed to understand whether cabozantinib preferentially inhibits the cell growth of human RCC cells under conditions of sunitinib resistance. To this end, we mimicked a TKI-based sequence treatment in vitro using sunitinib-resistant 786-O/S and Caki-2/S cells. Both cell lines were established following long-term treatment of 786-O and Caki-2 cells with sunitinib for a period of approximately four months. At this point, the cell viability of the 786-O/S and Caki-2/S cells under sunitinib treatment (3 µM, IC_50_) reached 80 and 95%, respectively, resulting in values significantly higher than those for the corresponding age-matched untreated 786-O/WT and Caki-2/WT cell lines (unpublished data, Supplementary Figure S1). To further test the effect of cabozantinib treatment, 786-O/S and Caki-2/S cells were cultivated as a monolayer under conditions of sunitinib withdrawal for 5 days. On the one hand, this period allowed us to remove the previous drug from the culture medium; on the other hand, it allowed us to retain the molecular characteristics of acquired resistance. Therefore, we can assert that the effects observed in the assays were attributable to cabozantinib, not to the previous medication. Both sunitinib-resistant cells and the corresponding untreated cells were growth-inhibited by 72-h exposure to increasing concentrations of cabozantinib ranging from 0 to 25 µM (Figure 1a, b) in a dose-dependent fashion. As shown by the dose–response curve, the 786-O/S cells were less growth-inhibited by cabozantinib than 786-O/WT cells, particularly by 13 and 15 µM doses of cabozantinib (*p*-value = 0.03 and 0.02, respectively). The drug reduced the cell viability of the 786-O/S cells to 50% (IC_50_) following exposure to 13 µM (±0.4), which is a greater reduction that that observed in 786-O/WT cells with an IC_50_ value of 10 µM (±0.6) *(p*-value = 0.03) (Figure 1a). We also observed a differential cell-line-specific sensitivity to cabozantinib, as Caki-2 cell lines showed less response to cabozantinib. In both Caki-2/WT and Caki-2/S cell lines, cell viability reached a plateau at 20 µM, after which it barely decreased below 40% (Figure 1b). Therefore, we implemented an “absolute IC_50_ calculation” (the concentration that provokes a response halfway between a blank and the full drug inhibition value) using a positive control that showed the maximum response to cabozantinib (786-O). We considered this value as a constant to constrain the baseline of the dose–response curve and determined the point on the curve where a 50% inhibition was observed. The absolute IC_50_ values were 14.5 µM (±1.50) and 13.6 µM (±1.05) for the Caki-2/WT and Caki-2/S cell lines, respectively (*p*-value = 0.6 versus Caki-2/WT) (Figure 1b). Overall, cabozantinib treatment inhibited the viability of the 786-O and Caki-2 cell lines when a concentration greater than 5 µM was applied. Preferential growth inhibition of sunitinib-resistant cell lines was not archived by cabozantinib treatment. On the contrary, we observed that long-term pretreatment with sunitinib might reduce the response to cabozantinib in 786-O/S cell lines, whereas no difference was observed between the Caki-2/WT and Caki-2/S cell lines. Our results indicate a cell-line-specific drug sensitivity between the 786-O and Caki-2 cells, with the latter showing low drug response values. Accordingly, we further studied the molecular differences that may contribute to the differential response of RCC cell lines to therapy.

### 2.2. Effect of Cabozantinib on Signaling Transduction in Sunitinib-Pretreated and Treatment-Naïve 786-O Cell Lines

**Figure 2:**
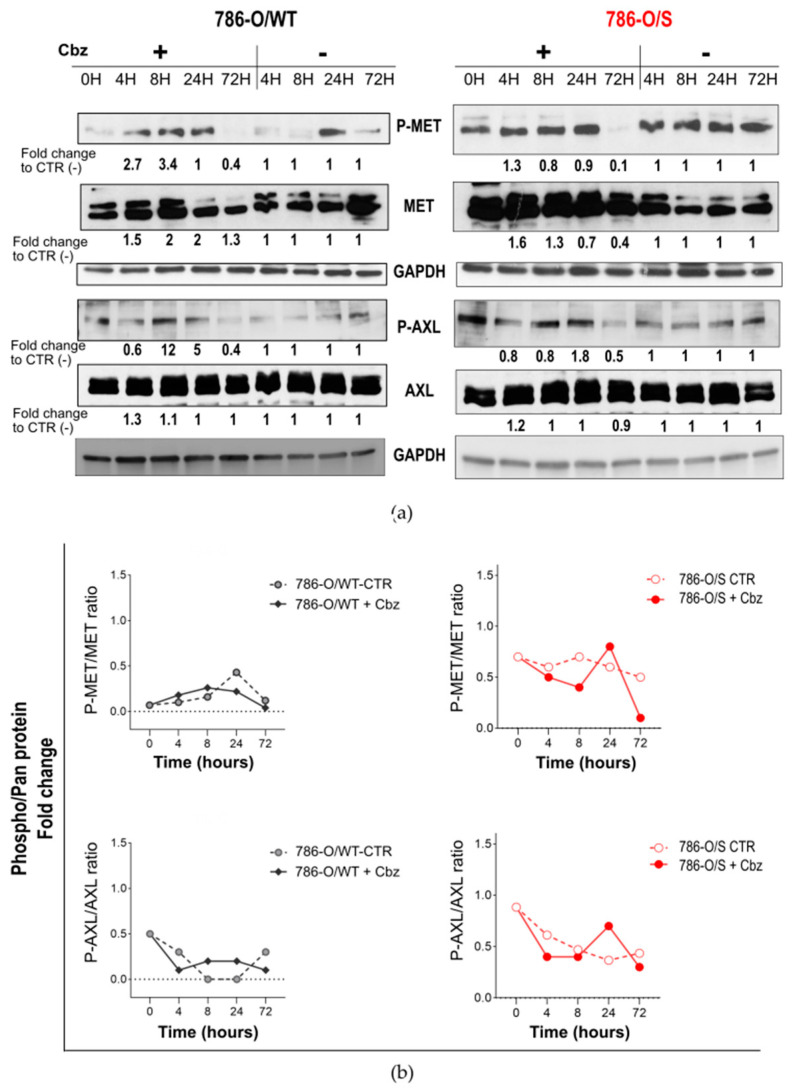
Effect of cabozantinib on signal transduction in human RCC treatment-naïve 786-O/WT cells and following long-term treatment with sunitinib in 786-O/S cells. (**a**) Cell lysates (20 µg) obtained from 786-O/WT and 786-O/S cells treated (+) with 12 µM of cabozantinib (Cbz) or untreated (−) with DMSO 0.1% at four time points were analyzed with an immunoblotting assay for specific antibodies with phosphorylated and total MET and AXL receptor proteins. GAPDH expression was selected as the loading control of samples for indicated proteins. The optical density (OD) of the area of each band was quantified with ImageJ-1.52a software and normalized to that of GAPDH. Numbers below each line represent the fold changes of the target proteins, which were obtained by dividing the normalized expression from each lane by the normalized expression of the relative untreated controls (set as 1). (**b**) Kinetics of MET (Y1234/5) and AXL (Y779) following treatment with cabozantinib (+Cbz) and in the untreated control (CTR) at indicated time points. Phosphorylation kinesis adjusted for total MET (P-MET/tot MET) and total AXL (P-AXL/tot AXL). Representative immunoblotting image and relative quantification. Full-length blots of independent duplicates are provided in Supplementary Figures S2, S5 and S6. The figure was generated with Inkscape (version 1.0.2, RRID: SCR_014479).

The effects of cabozantinib on MET and AXL receptors and intracellular signaling transduction (Figure S11) were analyzed in 786-O/WT and 786-O/S cell lines (Figure 2). We examined and compared the kinetics of the receptor tyrosine kinases MET and AXL, measured as phosphorylation states (P-MET and P-AXL), with and without 12 µM of cabozantinib for short-term (4–8 h) and extended periods of time (24 and 72 h). Such concentrations were chosen as average IC_50_ values of cabozantinib in all cell lines, allowing us to treat the cells with the same drug concentration. Overall, the sunitinib-resistant 786-O/S cell lines displayed a four- and two-fold higher constitutive level of P-MET and P-AXL, respectively, as well as an enhanced phosphorylation rate (phosphorylate/pan forms) compared with the treatment-naïve 786-O/WT cell lines (Figure 2b). Following treatment with cabozantinib, we did not observe a decrease in P-MET or P-AXL levels in the naïve 786-O/WT cell line; instead, we observed overexpression following short-term drug exposure to cabozantinib (Figure 2, left).

**Figure 3:**
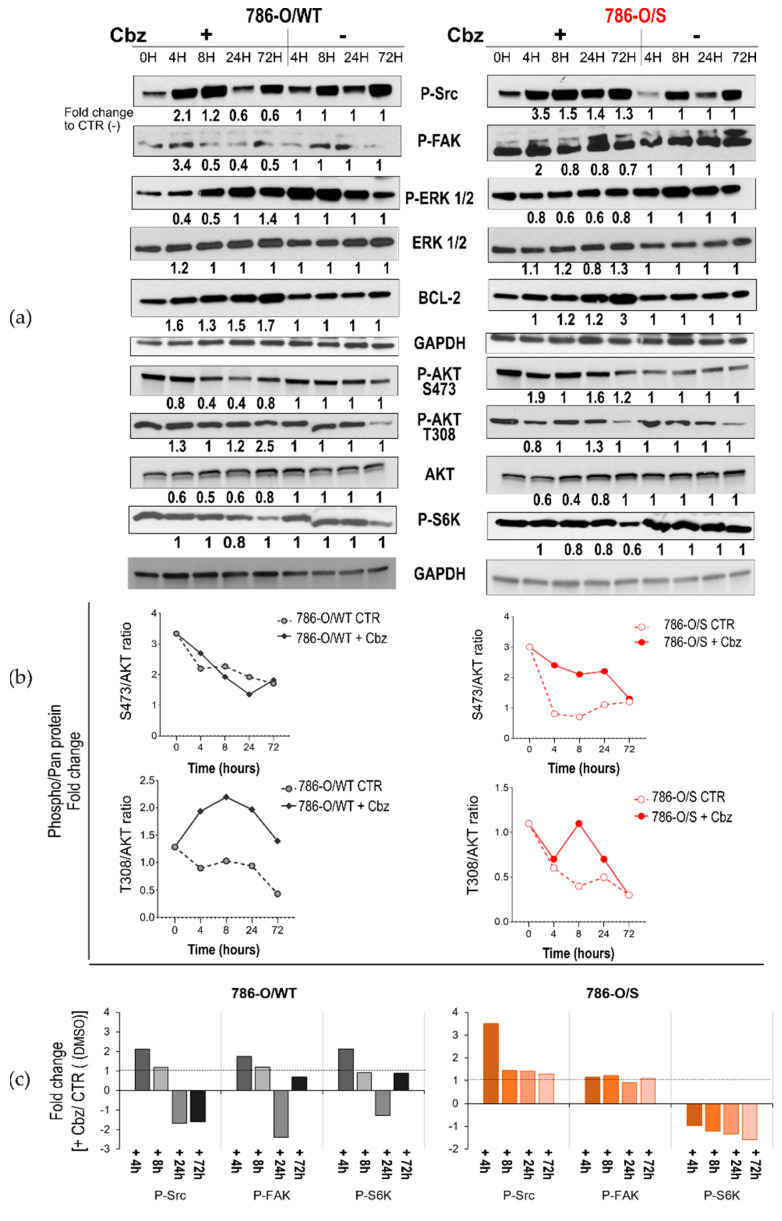
Effect of cabozantinib on signal transduction in human RCC treatment-naïve 786-O/WT cells and following long-term treatment with sunitinib in human RCC 786-O/S cell lines. (**a**) Cell lysates (20 µg) obtained from wild-type 786-O/WT and 786-O/S cells treated (+) with 12 µM of cabozantinib (Cbz) or untreated (−) with DMSO 0.1% at four time points were analyzed with an immunoblotting assay with specific antibodies for indicated proteins. GAPDH expression was chosen as the loading control for the samples. The optical density (OD) of the area of each band was quantified with ImageJ 1.52a software and normalized to that of GAPDH. Numbers below each line represent the fold changes of the target proteins, which were obtained by dividing the normalized expression from each lane by the normalized expression of the relative untreated controls (set as 1). (**b**) Kinetics of AKT (S473 and T308) in 786-O/WT and 786-O/S cells following treatment with cabozantinib (+Cbz) and untreated control (CTR) at indicated time points. The optical density (OD) of the area of each band of phosphorylated and total AKT was quantified with ImageJ 1.52a software and normalized to that of GAPDH. Phosphorylation kinesis adjusted for AKT (S473/tot AKT and T308/tot AKT). (**c**) Comparative expression levels (fold change) of proteins P-Src, P-FAK and P-S6K between cabozantinib-treated and untreated samples. The relative protein expression (densitometry normalized to the loading control) of each cabozantinib-treated sample was compared with the corresponding DMSO-treated (control, CTR) samples (set as 1) to assess the fold change between the two experimental conditions. Representative immunoblotting image and relative quantification. Full-length blots of independent duplicates are presented in Supplementary Figures S3–S6. The figure was generated with Inkscape (version 1.0.2, RRID: SCR_014479).

Short-term drug incubation elicited the total form of MET (increased in both phosphorylated and total levels), in addition to inducing AXL phosphorylation without changing its total protein level. In contrast to the 786-O/WT cell line, the high constitutive phosphorylation level of MET and AXL was not induced further by cabozantinib treatment in the 786-O/S cells at the same time points (Figure 2b). Changes in the activity of the two receptors were observed only after 72 h of drug exposure in both 786-O/WT and 786-O/S cell lines, when the phosphorylation decreased but not the total form, implying that sunitinib-resistant 786-O/S cells are phenotypically different from their 786-O/WT counterparts in terms of MET and AXL expressions. Such a difference might affect—to a certain extent—the effects of cabozantinib on P-MET and P-AXL modulations in the 786-O cell lines. This result also suggests that cabozantinib might act via other targets. A preliminary study on hepatocellular carcinoma showed that the focal adhesion kinase protein (FAK) might be a regulator of the drug response to anti-MET therapy [40]. A five-fold increase in the basal level of phosphorylated P-FAK and its upstream activator, the steroid receptor coactivator (Src) tyrosine kinase, was associated with sunitinib resistance in 786-O/S cell lines (Figure 3a). Cabozantinib reduced the levels of P-Src and P-FAK 24 h after treatment in 786-O/WT cells (fold change = −1.7 and −2.5, respectively), but no change in the protein expression was observed in the corresponding sunitinib-resistant cell lines. We further analyzed signaling proteins, including the full activation of protein kinase B (or AKT), which was determined by phosphorylation at serine site 473 (S473) and threonine site 308 (T308). Exposure to cabozantinib caused the downregulation of P-AKT (S473) only in the 786-O/WT cell line 8 and 24 h after treatment (fold change = −2.5). In contrast, cabozantinib induced the complete activation of AKT in the 786-O/S cell line. Considering that the level and activity of the kinase generally affect the amplitude of the signal pathway, we deduced that high levels of P-Src/P-FAK and AKT proteins may support a strong kinase signal despite cabozantinib treatment. Finally, cabozantinib caused the slight downregulation of kinase P-S6K (fold change = −1.8) only in the sunitinib-resistant 786-O/S cells 72 h after treatment (Figure 3a). We also described the regulation of signaling proteins that were equally affected by cabozantinib between 786-O/WT and 786-O/S cell lines, including a time-dependent upregulation of the antiapoptotic protein BCL-2 and a de-phosphorylation of ERK1/2 following short-term cabozantinib treatment. The latter was most evident in the treatment-naïve 786-O/WT cell line, with a reduction of 60% vs. 40% in the 786/S cells. These data indicate that cabozantinib, as a multityrosine kinase inhibitor, may act through other molecular mechanisms in addition to its principal molecular targets, MET and AXL, the inhibitions of which were weak in 786-O cells. Moreover, the differential modulation of the abovementioned mechanisms between the treatment-naïve (786-O/WT) and sunitinib-resistant (786-O/S) cell lines might be reliant on sunitinib resistance, suggesting a diverse phenotype among the two cell lines.

### 2.3. Effect of Cabozantinib on Signaling Transduction in Sunitinib-Pretreated and Treatment-Naïve Caki-2 Cell Lines

**Figure 4:**
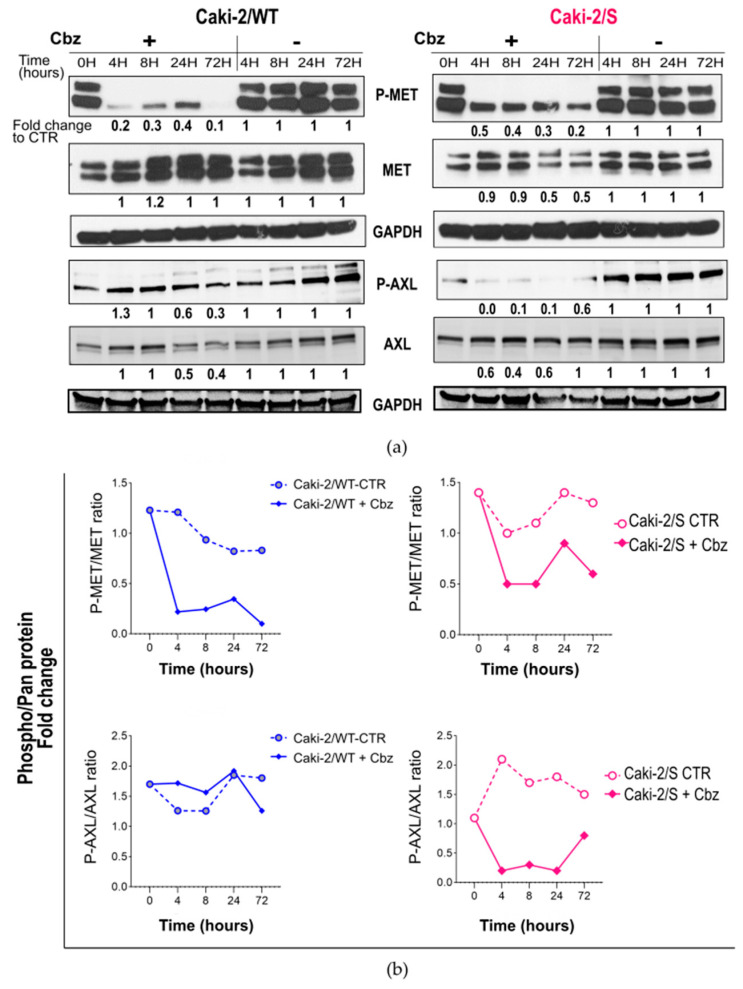
Effect of cabozantinib on signal transduction in human RCC treatment-naïve Caki-2/WT cells and following long-term treatment with sunitinib Caki-2/S cell lines. (**a**) Cell lysates (20 µg) obtained from Caki-2/WT and Caki-2/S cells treated (+) with 12 µM of cabozantinib (Cbz) or untreated (−) with DMSO 0.1% at four time points were analyzed with an immunoblotting assay for specific antibodies with phosphorylated and total MET and AXL receptor proteins. GAPDH expression was selected as the loading control of samples for indicated proteins. The optical density (OD) of the area of each band was quantified with ImageJ 1.52a software and normalized to that of GAPDH. Numbers below each line represent the fold changes of the target proteins, which were obtained by dividing the normalized expression from each lane by the normalized expression of the relative untreated controls (set as 1). (**b**) Kinetics of MET (Y1234/5) and AXL (Y779) following treatment with cabozantinib (+Cbz) and in the untreated control (CTR) at indicated time points. Phosphorylation kinesis adjusted for total MET (P-MET/tot MET) and total AXL (P-AXL/tot AXL). Representative immunoblotting image and relative quantification. Full-length blots of independent duplicates are presented in Supplementary Figures S2, S9 and S10. The figure was generated with Inkscape (version 1.0.2, RRID: SCR_014479).

For the Caki-2 cell lines (Figure 4), the dose–response curve assay presented reduced drug sensitivity to cabozantinib (Figure 1b), independent of pretreatment (in first-line treatment with sunitinib). We further investigated whether the response to the sunitinib > cabozantinib treatment sequence might differ from that to cabozantinib alone at the molecular level. It is usually believed that cell lines expressing high levels of the target receptor of a specific TKI present improved drug sensitivity values. However, no such relationship was observed concerning the P-MET protein and sensitivity to cabozantinib. Unlike the 786-O cells, both Caki-2/WT and Caki-2/S cell lines expressed a pro-MET protein band (at 175 kDa) and a high level of P-MET, which resulted in completely activated receptors (phosphorylated/pan MET = 1.3) (Figure 4a).

**Figure 5:**
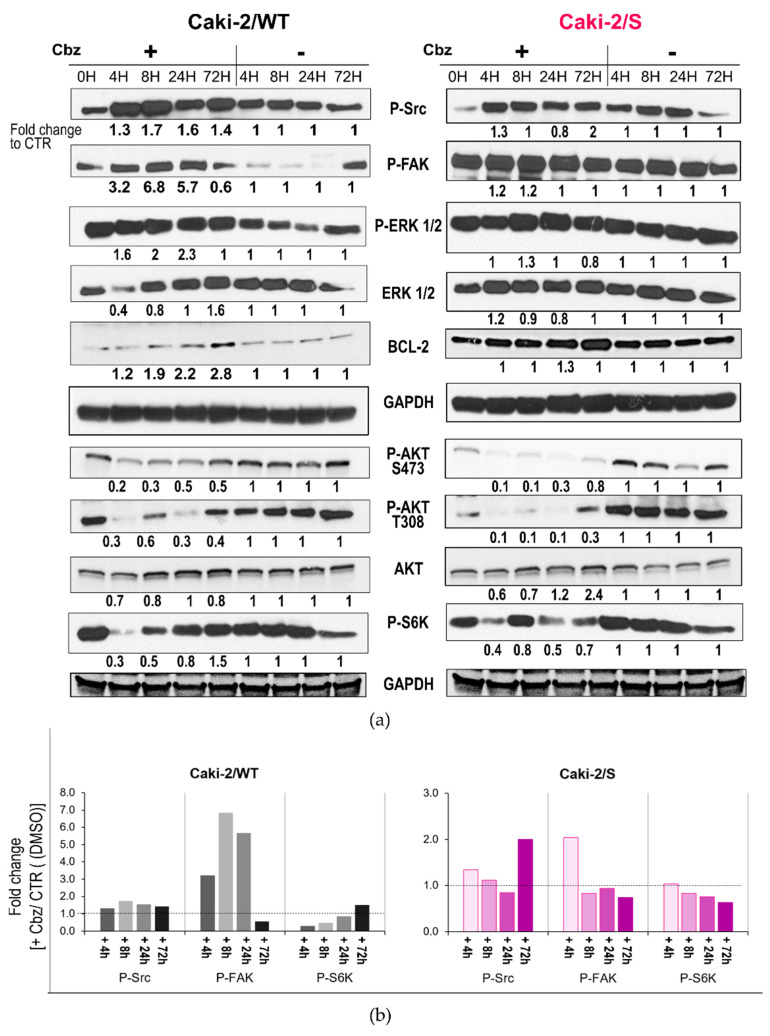
Effect of cabozantinib on signal transduction in human RCC treatment-naïve Caki-2/WT cells and following long-term treatment with sunitinib in Caki-2/S cell lines. (**a**) Cell lysates (20 µg) obtained from Caki-2/WT and Caki-2/S cells treated (+) with 12 µM of cabozantinib (Cbz) or untreated (−) with DMSO 0.1% at four time points were analyzed with an immunoblotting assay with specific antibodies for the indicated proteins. GAPDH expression was selected as the loading control of the samples. The optical density (OD) of the area of each band was quantified with ImageJ 1.52a software and normalized to that of GAPDH. Numbers below each line represent the fold changes of the target proteins, which were obtained by dividing the normalized expression from each lane by the normalized expression of the relative untreated controls (set as 1). (**b**) Comparative expression levels (fold change) of the P-Src, P-FAK and P-S6K proteins between cabozantinib-treated and untreated samples. The relative targeted protein expression (densitometry normalized to the loading control) of each cabozantinib-treated sample was compared with the corresponding DMSO-treated (control, CTR) samples (set as 1) to assess the fold change occurring under the two experimental conditions. Representative immunoblotting image and relative quantification. Full-length blots of independent duplicates are presented in Supplementary Figures S7–S10. The figure was generated with Inkscape (version 1.0.2, RRID: SCR_014479).

Cabozantinib strongly deactivated the receptor after 4 h and throughout all the following time points of treatment in both cell lines. However, the molecular response to the drug did not correspond to the functional response (cell viability). Similar to the 786-O/S cell line, high expression and activation of the AXL receptor were related to sunitinib resistance occurring in Caki-2/S cell lines. Cabozantinib dephosphorylated P-AXL exclusively in sunitinib-resistant cells. Moreover, we detected a decrease in the total forms of MET and AXL in the sunitinib-resistant Caki-2/S cells, suggesting that cabozantinib inhibits the total expression of the two RTKs and not exclusively their activity. Based on these data, we inferred that only the modulation of P-AXL activity depended on the sunitinib > cabozantinib sequencing treatment. Following long-term sunitinib treatment, the specific signaling proteins P-FAK and BCL-2 presented seven- and five-fold higher protein levels, respectively, in the Caki-2/S cell line in comparison with the treatment-naïve Caki-2/WT cell line (Figure 5). The treatment of Caki-2/WT cells with cabozantinib for short and prolonged incubation periods induced a time-dependent upregulation of the two proteins in the Caki-2/WT cells (P-FAK: fold change ranging from three to seven; BCL-2: fold change ranging from 1.2 to 3 vs. the untreated control). The same experimental conditions slightly enhanced the constitutively high expression of P-FAK and BCL-2 in Caki-2/S cell lines (Figure 5b). Both Caki-2 cell lines displayed a basal level of phosphorylated P-ERK ½, and cabozantinib further induced the kinase phosphorylation occurring during all time points of treatment, with no change in the total form of the protein. These data suggest that in the Caki-2 cell line, pretreatment with sunitinib has a limited impact on the activity of cabozantinib in inhibiting MET-dependent signaling proteins (Figure S11). Nonetheless, these transduction kinases were upregulated following exposure to cabozantinib. The activation of the AKT/mTOR signal is a hallmark of RCC and sunitinib resistance. Whereas cabozantinib strongly deactivated AKT signaling (S473 and T308) in Caki-2/WT and Caki-2/S cells, the drug preferentially inhibited the expression of kinase P-S6K, with a two-fold change in the Caki-2/S cells (similar to the 786-O/S cell lines). This result indicates that pretreatment with sunitinib might support cabozantinib in inhibiting the signal involving the mTOR pathway. Overall, the cell survival values mirror the results of the cell signaling analysis. The 786-O and Caki-2 cells heterogeneously responded to cabozantinib not only at the cellular level, but also with respect to the modulation of signal transduction proteins, such as ERK, AKT and FAK. Although cabozantinib reduced viability in a dose-dependent manner, a lower drug sensitivity value was observed in the Caki-2/WT cell line and the sunitinib-resistant 786-O/S and Caki-2/S cells. This behavior was reflected in differential modulations of the MET, AXL and Src-FAK properties, as well as AKT signaling proteins.

## 3. Discussion

The aberrant activation of receptor tyrosine kinases MET and AXL, or MET amplification, is a major cause of the poor prognosis of RCC, as they are potent activators of cell proliferation and survival pathways and the development of bone metastases; most importantly, their activity helps cancer cells to bypass the growth-inhibitory effect of VEGFR-TKI sunitinib [41,42,43,44,45]. The multitargeted TKI cabozantinib has shown antitumor activity against tumor cells expressing activated (phosphorylated) receptors MET and AXL (P-MET and P-AXL) [46,47], including sunitinib-resistant human RCC cells [38]. Studies have provided a preclinical rationale for the development of MET and AXL inhibitors in resistance or transient insensitivity to antiangiogenic therapy [48]. This evidence suggests that cabozantinib might preferentially inhibit the growth of sunitinib-resistant cells compared with their sensitive counterparts. Clinical data also demonstrate that the use of this drug is an effective therapeutic strategy for mRCC-pretreated patients who display disease progression following first-line TKI therapy, such as sunitinib treatment [49,50]. However, the paucity of preclinical studies and the absence of validated molecular or clinical predictors in this patient population represent a key challenge for uro-oncologists. Describing the potential consequences of long-term pretreatment with sunitinib for cell sensitivity to the subsequent use of cabozantinib was the aim of our study. This issue is of particular importance because optimizing the treatment sequence could potentially delay or hamper the unavoidable emergence of resistance to targeted therapy [27,29,51,52]. We exposed treatment-naïve (wild type) 786-O/WT and Caki-2/WT cell lines and the paired sunitinib-resistant 786-O/S and Caki-2/S sublines to cabozantinib to understand the molecular mechanisms of response to therapy alone and sequential TKI-based treatment.

The human RCC cell lines used in this study, 786-O/WT and Caki-2/WT, displayed a heterogeneous response to cabozantinib, with Caki-2 found to be less responsive to treatment. This result may have been caused by the different biological backgrounds of these cells, although a *VHL* gene mutation was reported for both cell lines (https://www.depmap.org (release 22Q4 (accessed on 7 December 2022)) [53,54]. Caki-2/WT cells also presented a constitutive activation (rate of protein phosphorylation) of MET and AXL, unlike the 786-O/WT cells. These initial observations emphasize the variable response to treatment among patients, which may rationalize the use of biomarker-based approaches. For instance, the recent multiregion exosome sequencing of several RCC patients revealed intra- and intertumor heterogeneity [55,56], which influences clinical progression, treatment outcomes and treatment decision making. It should be noted that long-term pretreatment using sunitinib in the 786-O/S and Caki-2/S cell lines elicited a higher degree of AXL phosphorylation, whereas P-MET was significantly upregulated only in the 786-O/S cell line. Despite sunitinib withdrawal, P-MET and P-AXL remained overexpressed in sunitinib-resistant cells. This suggests a possible MET and AXL addiction. These results are consistent with those obtained by Zhou and colleagues, who reported that AXL and MET are involved in acquired resistance to sunitinib in RCC cell lines [38]. Based on this evidence and on the results of preclinical studies [16,38,57], we hypothesized that the overexpression and activation of the two receptors may preferentially facilitate the inhibitory effect of cabozantinib on the growth of 786-O/S and Caki-2 cells. However, we were not able to confirm this completely. At the molecular level, the response of the tested cells to cabozantinib depended on two main factors: the drug exposure time and the cell line/cell phenotype. We observed that only a short exposure time to cabozantinib enhanced the low basal level of P-MET and P-AXL in 786-O/WT cell lines (approximately 20% of receptor phosphorylation), which can be explained by the effect of low initial drug cytotoxicity levels. On the contrary, short and prolonged drug exposure time (up to 24 h) against the resistant 786-O/S cell line did not affect the activation of P-MET and P-AXL. Overall, these findings indicate that the activation of the two receptors is important to protect cancer cells from cabozantinib-induced cell death; however, the activation of these receptors may also improve the survival of 786-O/S cells when prolonged cabozantinib incubation time is applied. As the results show, the sunitinib-pretreated 786-O/S cell line tolerated cabozantinib better than the corresponding treatment-naïve cells following 72 h of exposure to the cell viability WST-1 assay. Moreover, Caki-2 cells are a possible MET- and AXL-driven phenotype, independent of chronic treatment with sunitinib. Whereas cabozantinib mainly abolished MET activation, the drug had little effect on cellular viability, as a higher drug concentration (22 µM) decreased cell vitality by only 40%. Cabozantinib concentrations higher than 25 µM led to drug precipitation in a crystalline form due to the property of poor aqueous solubility. Therefore, we considered Caki-2 as a “cabozantinib low-responder” cell line because the cells were not considerably affected by the treatment in a dose-dependent manner. Different molecular mechanisms counteracting MET inhibition activity have been described in the literature [58] as a result of the feedback loops responsible for the regulation of drug targets in tumor cells. These include MET gene amplification and increased constitutive phosphorylation of the receptor following exposure to the MET inhibitor PHA-665752 in “MET-driven” gastric cancer cells [59], as well as compensatory EGFR/MET crosstalk, which has been implicated in tumorigenesis and resistance to MET inhibition in colorectal cancer and hepatocellular carcinomas (HCCs) [60,61]. In this regard, further investigations are required for RCCs. Moreover, AXL activity can promote the lateral activation of MET in RCC through the steroid receptor coactivator (Src) tyrosine kinase, a downstream signal transducer and regulator of RTKs in the intracellular domain [30]. These observations are in accordance with our results to some extent, as the overexpression of P-MET was accompanied by the activation of P-AXL and Src. Therefore, the involvement of MET and AXL in cabozantinib-induced cell growth inhibition is complex. On the one hand, cabozantinib induced a short activation time of MET and AXL in the 786-O/WT cell line, which presented a low constitutive level of the two activated receptors. Such an event can be explained as a pro-survival mechanism that clearly depends on the incubation time, as long-term exposure leads to the inactivation of receptors. On the other hand, the activated molecular state of MET and AXL did not improve cell responsiveness to cabozantinib and growth inhibition of Caki-2/WT, 786-O/S and Caki-2/S cell lines, nor can it be viewed as a predictable indicator of drug response activity. This may depend on the cell phenotype, as previously, and on other kinases required for cell survival. We observed that the activation of Src and focal adhesion kinase (FAK) counteracted the inhibitory effect of cabozantinib, mostly in the Caki-2/WT, Caki-2/S and 786-O/S cell lines. FAK, in association with Src, can be activated through phosphorylation and coordinate signals obtained from integrins [62,63] and receptor tyrosine kinases, which regulate cell proliferation and cellular motility [64,65]. Recent studies conducted on different tumor entities, such as breast cancer and HCC, indicated that MET and FAK are involved in the signaling crosstalk occurring in tumor cells [66,67], representing a mechanism of “oncogene switching”, which is adopted by cancer cells to counteract the inhibition of specific receptor tyrosine kinases [68]. Our data are supported by a study conducted by Wang and colleagues [69] describing increased FAK phosphorylation activity following cabozantinib treatment for HCC, which, in turn, limited the effect of the drug. Moreover, simultaneous exposure to the FAK inhibitor CT-707 abolished cabozantinib-induced FAK activation, implying the possible synergistic effect of combination therapy interfering with these two intracellular signaling proteins. Regarding the factor of drug resistance, several reports have proposed FAK and Src as players in therapy resistance in different tumor models [70,71,72]. Their downregulation increases the cytotoxicity of docetaxel in ovarian cancer [73], as well as their sensitivity to gemcitabine in pancreatic cancer [74]. A recent study presented the paradoxical cell growth evoked by TKIs through the activation of Src and FAK, which, in turn, initiates ERK signaling [75]. In addition, a study conducted on synthetic lethal screening [40] indicated that integrin α5, an upstream activator of FAK, is one of the key regulators related to drug resistance to anti-MET therapy. Assessing the protein level of integrin-dependent signaling was beyond the scope of our study. Further research is required to profile the possible crosstalk occurring between RTK and integrins in RCCs. Ultimately, Src and FAK might induce growth signals and predict potential treatment resistance to cabozantinib in our cell models. Moreover, the two kinases were induced in the CAKI-2/WT cell lines, but the extremely high basal phosphorylation activity of Src and FAK in both sunitinib-resistant cell lines did not change following their exposure to cabozantinib. This suggests that the differential modulation of the Src/FAK complex may depend on long-term sunitinib pretreatment and further sustain the survival and drug tolerance of the resistant cells. Finally, a closer observation of our results implies that the sunitinib > cabozantinib TKI sequence affects the P-S6K/mTOR signal. However, this phenomenon was observed only during late exposure to cabozantinib in 786-O/S and Caki-2/S cell lines.

Overall, the data presented in this study indicate that the high expression of the main target of cabozantinib, MET, does not reflect the drug-responsiveness property, as discussed in other studies. Considering that a viability assay, such as WST-1, evaluates the cell proliferation rate, cell size, metabolic rate and survival, it cannot be directly compared with the protein expression data. Nonetheless, we can argue that the cell-line-specific response to cabozantinib might be the result of the off-target effects of the drug. As a general observation, the variability occurring in cellular responses to cabozantinib between 786-O and Caki-2 cell lines suggests the preferential engagement of a specific downstream pathway. For instance, cabozantinib activated ERK and completely hampered AKT signaling at the T308 and S473 sites only in the Caki-2 cell line, and these events were independent of pretreatment with sunitinib. On the contrary, the cabozantinib treatment schedule (alone or in sequence) of the 786-O cell line may affect the AKT signaling process, which is one of the most important pathways involved in cell proliferation, survival and transformation occurring in RCC tumorigenesis [10,76], as well as acquired resistance [77]. We observed that AKT was fully activated (phosphorylation at site S473 and T308) following cabozantinib exposure only in 786-O/S cells. It is well-described in the literature that activated RTKs and phosphatidylinositol 3-kinase (PI3K) mediate protein phosphorylation at the catalytic site of AKT (T308), which is required for mTOR activation [76,78]. The phosphorylation state of the S473 residue not only represents a mechanism by which AKT recognizes its downstream substrates, but is also an indicator that the mTORC2 complex is active in 786-O/S [79,80]. mTORC2-mediated S743 phosphorylation might represent a mechanism adopted by cells to regulate glucose consumption during metabolic stress [81,82], or it might promote cytoskeleton reorganization and cell survival by upregulating antiapoptotic proteins in the BCL2 family [83]. Cabozantinib induced BCL-2 expression in all cell lines, but it was increased by three-fold in the 786-O/S cell line. The differential drug response between cell lines described in the present study could allow us to determine the molecular signature to define predictive response profiles to TKI treatment and treatment schedules. This study is subject to some limitations that can be addressed in future research. For example, additional methods such as cell cycle and apoptosis assays could provide a more detailed picture of specific cellular events that may be affected by TKI-based sequencing therapy. Moreover, these data need to be validated in a three-dimensional human spheroid and organoid cell culture due to the limited number of cell lines analyzed in this study. In this study, we did not analyze the effect of cabozantinib on its other target, RTK VEGFR. The functional significance of VEGFR in angiogenesis is well-described in the literature; however, we focused on the direct effect of TKIs on molecular targets that are responsible for cell proliferation, survival, and resistance in tumor cells.

Our results shed light on the in vitro effects of therapy sequencing for mRCCs. In summary, the upregulation of P-MET and P-AXL observed in our cell models did not suffice to sensitize cells to cabozantinib in the second-line treatment. The relationship between the cytotoxicity of cabozantinib, MET and AXL activities might depend on sunitinib pretreatment, drug incubation time and the cell phenotype. The data presented here also suggest a cell-line-dependent mode of action of cabozantinib, reflecting the heterogeneity among patients. Receptors MET and AXL might be activated by cabozantinib as a feedback mechanism only in 786-O. However, the sustained activation of the two receptors was preserved following prolonged drug exposure only in the sunitinib-resistant 786-O/S cell lines. On the contrary, the strong P-MET inhibition caused by cabozantinib was not affected by sunitinib pretreatment in the Caki-2 cell line, whereas P-AXL downregulation was affected by treatment with the sunitinib > cabozantinib sequence. In general, sunitinib pretreatment of both 786-O/S and Caki-2/S cells resulted in Src and FAK overexpression, ensuring a low sensitivity value for the second-line treatment.

## 4. Material and Methods

### 4.1. Compounds

Sunitinib malate was purchased from Tocris Bioscience (Bristol, UK), and cabozantinib was purchased from TargetMol Chemicals Inc. (Wellesley Hills, MA, USA). The compounds were dissolved in DMSO, stored as aliquots at −80 °C and thawed shortly before use. Sunitinib malate was used to prepare cell stocks of the sunitinib-resistant cell lines prior to the investigation of sequential TKI treatment.

### 4.2. Cell Culture

For the current study, human clear cell RCC 786-O and Caki-2 cell lines were purchased from the American Type Culture Collection (ATCC) (LGC Standards, Teddington, UK). These cell lines are both representative of subtype ccRCCs bearing a VHL mutation (the main feature of ccRCCs). The differences in the genetic backgrounds between the two cell lines were also reported, which helped us to include the patient heterogeneity effect in our study. The 786-O cell lines (mutant *TP53* and *PTEN* and wild-type *PBRM1*) (Broad Institute DepMap dataset, https://www.depmap.org (release 22Q4, accessed on 7 December 2022)) were cultivated in a 1:1 mixture of Dulbecco’s modified Eagle medium (DMEM) and RPMI-1640 (Sigma-Aldricht, Saint Louis, MO, USA), whereas the Caki-2 cells (mutant *PBRM1* and wild-type *PTEN* and *TP53*) (Broad Institute DepMap dataset, https://www.depmap.org (release 22Q4; accessed on 7 December 2022) were propagated in RPMI-1640, both containing 10% heat-inactivated fetal bovine serum (FBS). The cell growth conditions were set at 37 °C in a humid environment with 5% CO_2_. Exponentially growing cells were used for all experiments.

### 4.3. Establishment of Sunitinib-Resistant Cell Lines

To assess the activity of cabozantinib in the second-line setting following failed sunitinib treatment, we referred to a panel of sunitinib-resistant cell lines previously generated at our institution (data not published). Briefly, the IC_50_ (inhibitor concentration that causes 50% cell death) value of sunitinib was determined by a dose–response assay performed over 72 h and measured by a WST-1 cell viability test, as described below. To generate sunitinib-resistant cells lines, 786-O and Caki-2 cell lines were exposed to the selective pressure of sunitinib at their respective IC_50_ values (3 µM) for approximately four months and defined as 786-O/S and Caki-2/S cells, respectively. During the development stage, age-matched untreated wild-type (WT) 786-O/WT and Caki-2/WT cells were also subcultivated. The sunitinib tolerance of 786-O/S and Caki-2/S was routinely assessed and compared with their WT counterparts with a WST-1 test. In addition, cell stocks collected from both cell phenotypes were regularly cryopreserved. A stable high-sunitinib tolerance was achieved within 34 passages of the cell culture. The cell stocks corresponding to this passage were thawed for the experiments conducted in this study. Prior to performing sequential treatment with cabozantinib, the sunitinib-resistant cells were cultivated as a monolayer in medium without sunitinib for five days, along with the corresponding age-matched wild-type cell lines.

### 4.4. Cell Survival (WST-1) Assay

A WST-1 assay was performed to evaluate cell survival rates in response to cabozantinib exposure. Exponentially growing cells were used for the experiments, as well as age-matched untreated wild-type and sunitinib-resistant cells. Briefly, the untreated wild-type 786-O/WT and Caki-2/WT cell lines and their corresponding sunitinib-resistant cells, 786-O/S and Caki-2/S, were seeded in a 96-well plate at a density of 1 × 10^3^ or 2 × 10^3^/well, respectively, and allowed to adhere overnight at 37 °C. The cells were exposed to either DMSO (vehicle control, concentration of 0.25%) or increasing concentrations of cabozantinib (0.1–25 µM) for 72 h. Subsequently, a 10 µL/well of WST-1 solution (1:10 final dilution) was added, and the cells were incubated further at 37 °C for 60 min. The optical density was measured at 450 nm and a reference of 620 nm using a microplate reader (Tecan Infinite Pro- 200, Tecan Group Ltd., Maennedorf, Switzerland).

### 4.5. SDS-PAGE and Immunoblotting

For analysis of receptor tyrosine kinases and downstream intracellular signaling, the wild-type and age-matched sunitinib-resistant cells were seeded at a density of 2 × 10^5^ cells/mL in a 60 mm Petri dish and allowed to adhere overnight. On the following day, the cells were left untreated (vehicle control, 0.12%) or were exposed to 12 µM cabozantinib. This concentration roughly corresponds to the average IC_50_ value of cabozantinib in all cell lines that were analyzed, allowing us to treat the cells with the same drug concentrations. A previously conducted preclinical study showed a rapid inhibitory effect of cabozantinib on receptor MET phosphorylation within 8 h [16]. Therefore, we collected the cell lysate after 0-, 4-, 8-, 24- and 72 h time-point exposures to cabozantinib to analyze the drug’s effect on its targets and downstream signaling activity. At each time point, the cells were washed with ice-cold Dulbecco’s phosphate-buffered saline (MilliporeSigma, Burlington, MA, USA) and lysed in Tris-Triton buffer (50 mM Tris pH 7.4, 150 mM NaCl, CHAPS 1%, SDS 0,1%, Triton-X 1%) containing 1X protease inhibitor cocktail and 1X of phosphatase inhibitors (Roche/Merck) for 30 min on ice. The whole-cell lysates (WCLs) were clarified by centrifuging for 20 min at 12,000 rpm and 4 °C. Protein concentrations in WCLs were assessed with a Pierce^TM^ BCA protein assay kit (Thermo Scientific^TM^, Thermo Fisher Scientific, Waltham, MA, USA). Subsequently, 20 µg of cell lysates was separated by SDS-PAGE (Mini-PROTEAN electrophoresis cell, Bio-Rad Laboratories, Hercules, CA, USA) and transferred onto a methanol-activated polyvinylidene difluoride (PVDF) membrane (Serva Electrophoresis GmbH, Heidelberg, Germany) with a tank transfer system (Bio-Rad Laboratories, Hercules, CA, USA). The membranes were blocked in 5% blocking solution for 1 h at room temperature (RT). They were incubated further overnight with primary antibodies against PAN-MET, phospho-MET^Y1234/5^, PAN-AXL, phospho-AXL^Y779^, phospho-ERK1/2^T202/Y204^, PAN-AKT, phosphor-Akt^T308^, phosho-Akt^S473^, phosphoS6^S235/6^, phosho-FAK^Y397^ (focal adhesion kinase), phosphor-Src family, GAPDH (all antibodies were purchased from Cell Signaling Technology, NEB, Hitchin, UK) and BCL-2 (BD Bioscience, Franklin Lakes, NJ, USA) under agitation at 4 °C overnight. The membranes were then washed three times in Tris-buffered saline and Tween 20 (TBST) and incubated with horseradish peroxidase (HRP)-conjugated secondary antibodies (Cell Signaling Technology, NEB, Hitchin, UK) for one hour at room temperature (RT). Antibody detection was performed using an ECL-plus HRP substrate kit (Thermo-Scientific, Waltham, MA, USA). We performed a stripping and reprobing membrane protocol to visualize different proteins on the same membrane. Briefly, the membranes were agitated for 30 min in 25 mM glycine-hydrochloric acid (HCL), 1% sodium dodecyl sulfate (SDS) and pH 2 buffer at 50 °C. Subsequently, the blots were washed in 1X phosphate-buffered saline (PBS), equilibrated in Tris-buffered saline and 0.1% Tween 20^®^ detergent (TBST) and incubated with 5% blocking solution prior to reprobing with another antibody.

### 4.6. Statistical Analysis

Dose–response assays and IC_50_ values were analyzed with a nonlinear regression model using GraphPad Prism version 9.2.0 for Windows, GraphPad Software (San Diego, California USA, www.graphpad.com, accessed on 7 December 2022), and the *drc* packagev3.0-1 [84]. If the tested drug was not able to induce a full inhibition of cell viability (i.e., ≥40% relative to the vehicle control), we applied a fitting dose–response curve to obtain the absolute IC_50_. We calculated the concentration that provoked a response halfway between the blank (DMSO vehicle control, maximum measured response/cell viability) and a positive control (cells with a full inhibited response) using Graph Pad Prism^®^ software. Analysis of variance (ANOVA) and Student’s t-test were applied for comparative analysis with IBM^®^ SPSS^®^ Statistics for Windows (Version 27.0). *p*-values of ≤0.05 were considered statistically significant. The data obtained from all the experiments are presented as mean ± standard error (SE) of biological replicates, each derived from technical replicates. Graphs were generated with GraphPad Prism or in Excel. Composite images for publication were generated with Inkscape (version 1.0.2, RRID: SCR_014479). ImageJ 1.52a image analysis software (National Institutes of Health, Bethesda, MD, USA, https://imagej.nih.gov/ij, accessed on 7 December 2022) [85] was used to analyze and compare protein density values following immunoblotting. The values were determined as the optical density intensity (ODI) relative to the loading control. Protein abundance was expressed as the fold change of the difference between cabozantinib-treated samples and untreated controls. Protein abundance was expressed as a fold change of the difference between cabozantinib-treated samples and untreated controls by dividing the normalized expression from each lane by the normalized expression of the corresponding untreated controls.

## 5. Conclusions

Our in vitro study suggests that cabozantinib can efficiently primarily target P-MET and, to a limited extent, P-AXL in RCC. Despite a dose-dependent reduction in cellular viability following cabozantinib treatment, inferior drug sensitivity was observed in Caki-2/WT cells and sunitinib-resistant Caki-2/S and 786-O/S cell lines. Cabozantinib can induce intracellular signals involved in cell proliferation and survival processes and can also be considered an early indicator of response to therapy. The effect of cabozantinib on MET and MET-dependent signaling was highly variable between the two cell lines, highlighting the often-observed heterogeneity of patient sensitivity and responses to targeted therapy, which might complicate treatment standardization for all patients. Based on our analysis, we determined that the sequence of sunitinib followed by cabozantinib is not superior to first-line therapy with cabozantinib. In the second-line treatment, the MET- and AXL-driven status did not improve cell responsiveness to cabozantinib.

## Data Availability

The data presented in this study are available in tables in the Supplementary Materials and, upon request, from the corresponding authors.

## Supplementary Materials

The following supporting information can be downloaded at: https://www.mdpi.com/article/10.3390/ijms24065648/s1.

## Abbreviations

AKT: protein kinase B; ERK: extracellular signal-regulated kinase; FAK: focal adhesion kinase; IC50: half-maximal inhibitory concentration; ODI: optical density intensity; P-AXL: phosphorylated AXL; P-MET: phosphorylated MET; RCC: renal clear cell carcinoma; RTK: receptor tyrosine kinase; Src: proto-oncogene tyrosine-protein kinase; TKI: tyrosine kinase.
