# Supplementary material for: An In Vitro Analysis of TKI-Based Sequence Therapy in Renal Cell Carcinoma Cell Lines
Source: Int J Mol Sci. 2023 Mar 15;24(6):5648. doi: 10.3390/ijms24065648 (PMC10058472; doi:10.3390/ijms24065648)
Supplement: Supplementary file 1 [file ijms-24-05648-s001.zip › ijms-2199303-supplementary.pdf]

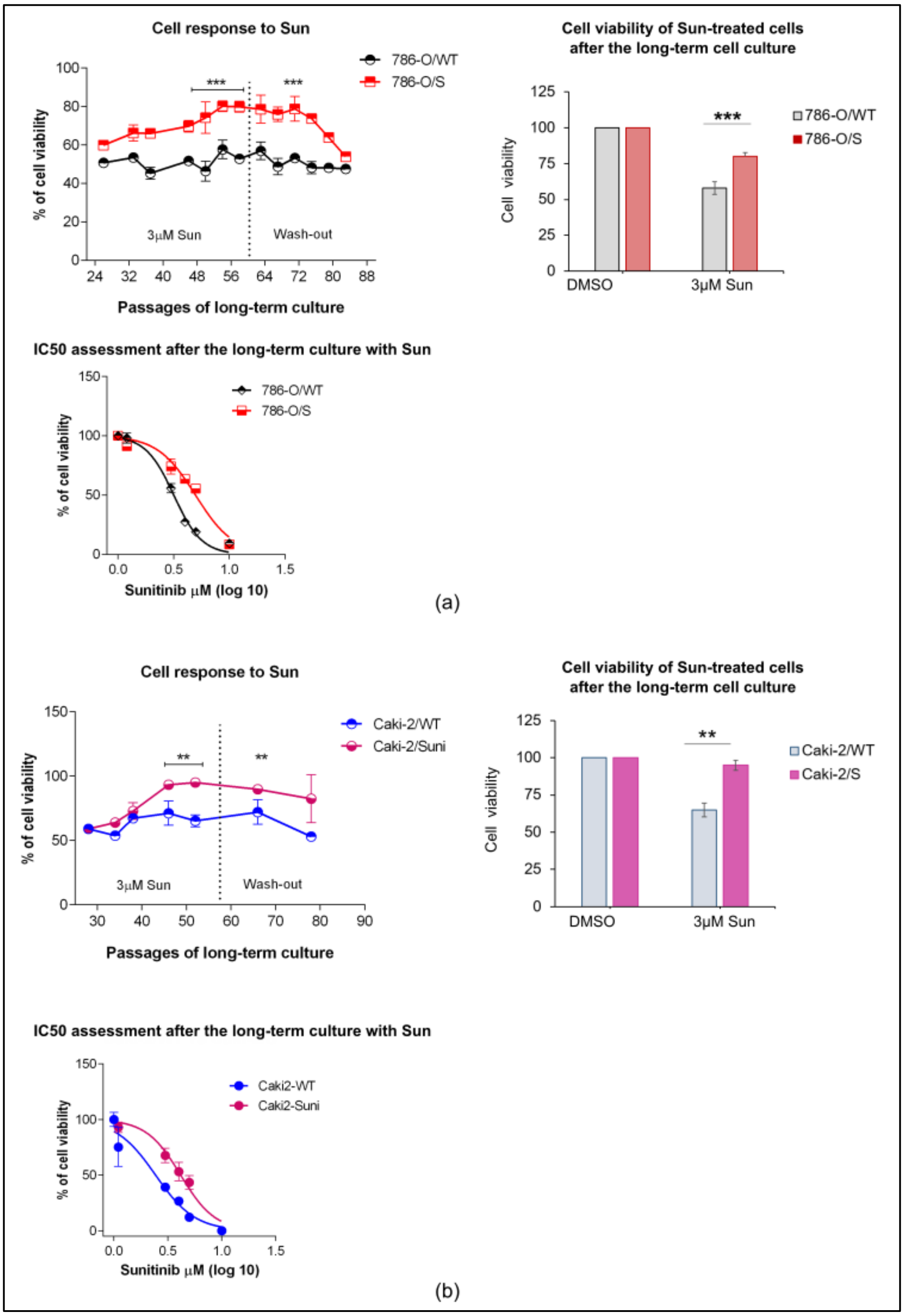

Figure S1. Development of drug tolerance towards sunitinib in 786-O/S (a) and Caki-2/S (b) cell lines. The 786-O and Caki-2 cells were exposed to the sunitinib IC_{50} concentration (3μM) for 72 hours. The surviving clones were re-seeded and further subjected to the drug twice a week. Changes occurring in the drug-tolerance values were routinely monitored using a cell viability assay (WST-1). Cell viability was calculated as a percentage relative to the DMSO (vehicle control). The results are presented as average values of three biological replicates ± SEM, ****p*-value < 0.001, as compared to the treatment-naïve, age-matched, wild-type cells exposed to sunitinib treatment for 72 hours. Following chronic exposure to sunitinib for a period of four months, the cell viability of both 786-O/S and Caki-2/S cell lines displayed a significant increase in cell viability when exposed to sunitinib (3μM). The IC_{50} concentrations were re-assessed for each cell line using a dose–response curve. The IC_{50} concentration of sunitinib was set to 5 μM (±0.19) and 5.11 μM (±0.12) compared to 3 μM in the paired 786-O/WT Caki-2/WT cells lines, respectively (*p*-value < 0.001).

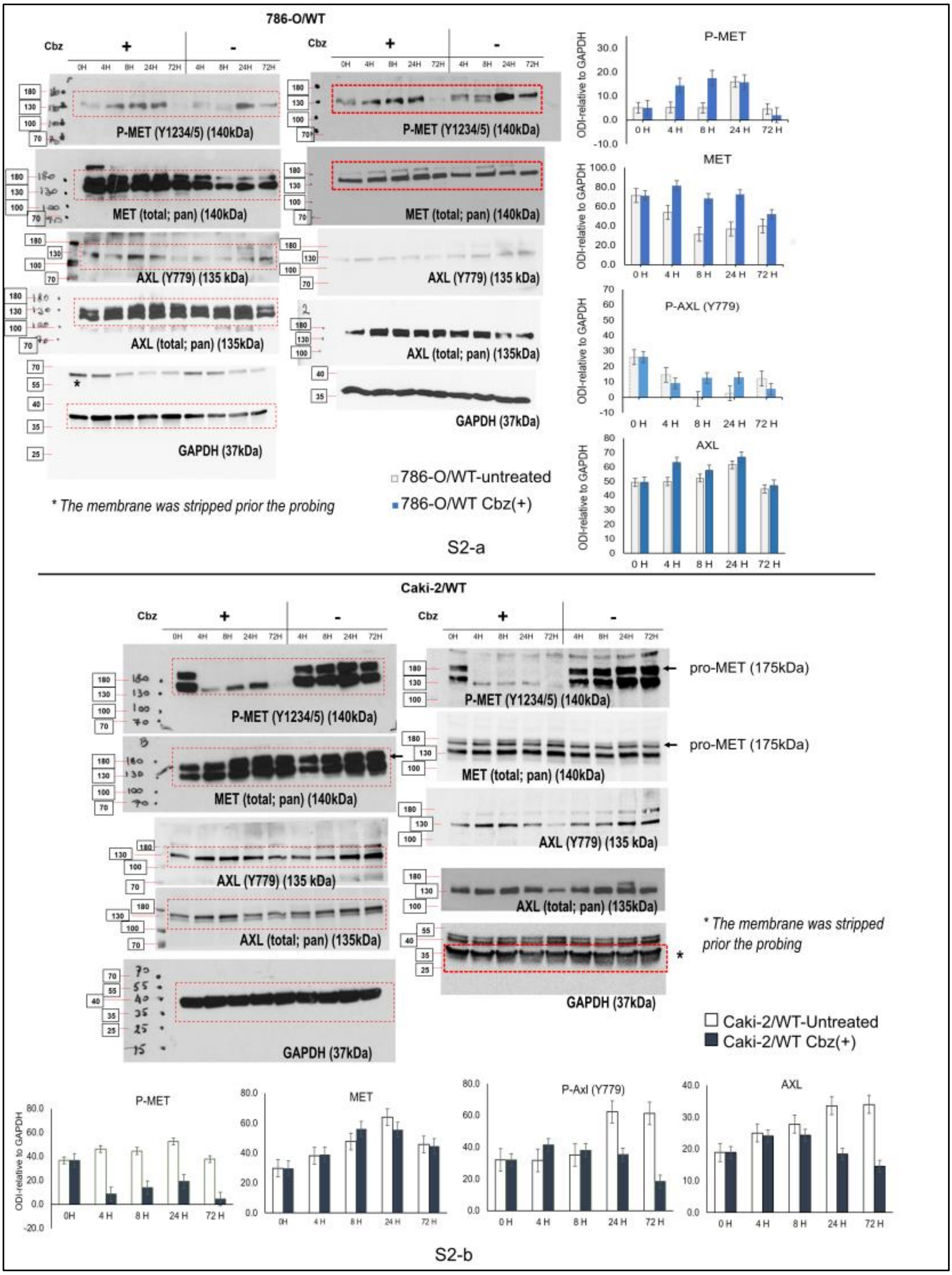

Figure S2. Effect of cabozantinib on receptor tyrosine kinases MET and AXL in treatment-naïve human RCC 786-O/WT and Caki-2/WT cell lines. Original immunoblotting results referring to Figures 2 and 4. Cell lysates obtained from wild-type 786-O/WT and Caki-2/WT cells treated (+) and untreated (-) with 12 μM of cabozantinib (Cbz) at four time-points were analyzed with an immunoblotting assay with specific antibodies for the indicated proteins. GAPDH expression was selected as the loading control of the samples. The densitometry readings of the band's intensity were performed with ImageJ software. The data evaluations, normalizing data representations, were performed using Microsoft Excel. Briefly, the pixel intensity of the target proteins and their backgrounds, alongside their loading control values, were inverted to binary values. Subsequently, the optical density (OD) of the target protein bands and loading controls were calculated by deducting the background to achieve high accuracy values. Finally, the ODI of the target protein was divided by the ODI of the loading control GAPDH.

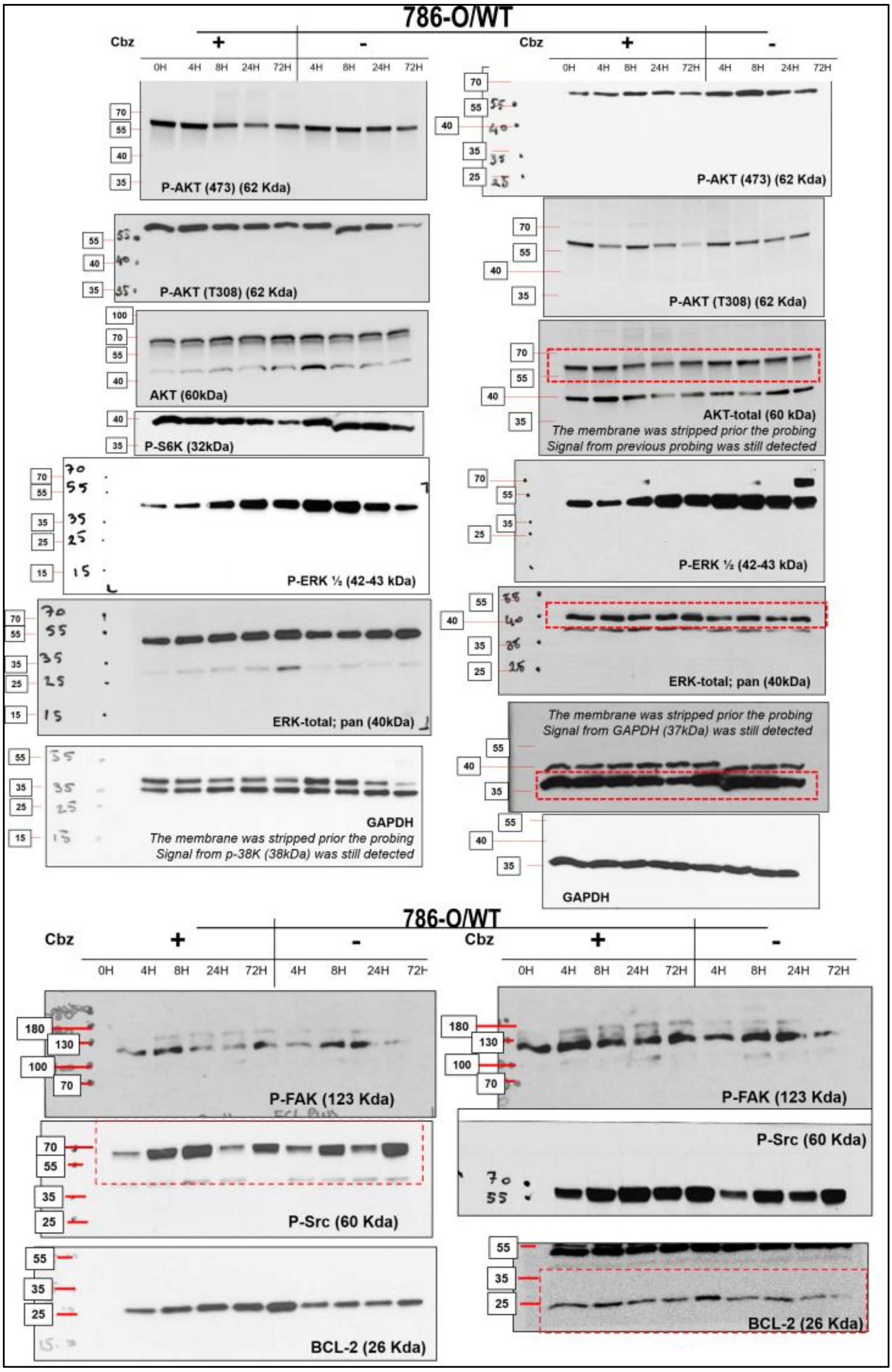

Figure S3. Effect of cabozantinib on signal transduction in treatment-naïve human RCC 786-O/WT cell lines. Original immunoblotting results referring to Figure 3. Cell lysates obtained from wild-type 786-O/WT cells treated (+) and untreated (-) with 12 μM of cabozantinib (Cbz) at four time-points were analyzed with an immunoblotting assay with specific antibodies for the indicated proteins. GAPDH expression was selected as the loading control of the samples. A stripping and re-probing protocol was conducted to remove the antibodies from the membranes and re-probed with another antibody.

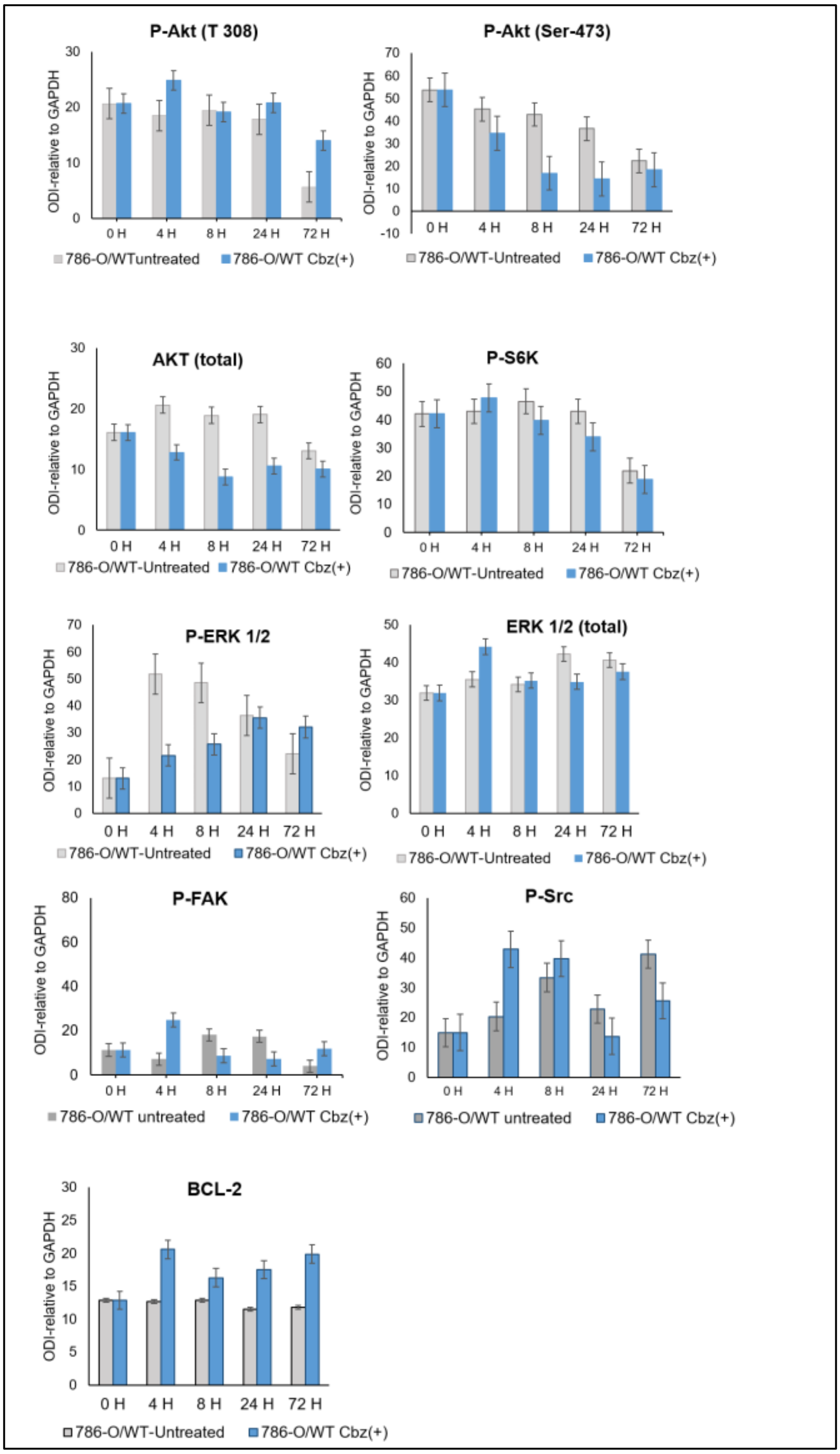

Figure S4. Quantification of Western blotting to analyze the effect of cabozantinib on signal transduction occurring in treatment-naïve human RCC 786-O/WT cell lines. Densitometry analysis referring to Figure 3 and Supplementary figure S3. The densitometry readings of the band's intensity were performed using *ImageJ* software [1]. The data evaluations, normalizing data representations, were performed using Microsoft Excel. Briefly, the pixel intensity of the target proteins and their backgrounds, alongside their loading control values, were inverted into binary values. Subsequently, the optical density (OD) of the target protein bands and loading controls were calculated by deducting the background to achieve high accuracy values. Finally, the ODI of the target protein was divided by the ODI of the loading control GAPDH.

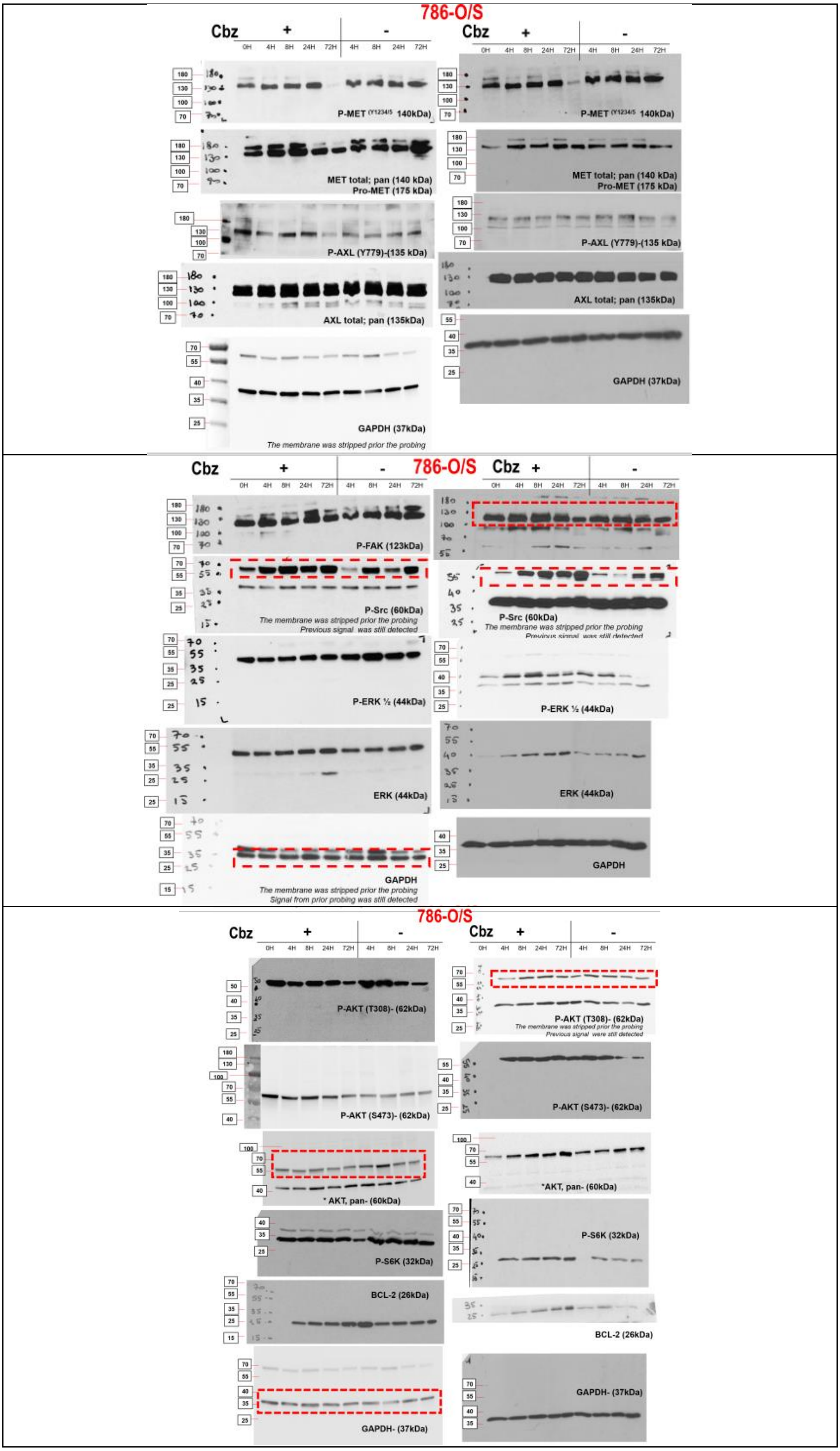

Figure S5. Effect of cabozantinib on receptor tyrosine kinases MET, AXL and the downstream signal transduction in the sunitinib-resistant 786-O/S cell line. Original immunoblotting results referring to Figures 2 and 3. Cell lysates obtained from wild-type 786-O/S cells treated (+) and untreated (-) with 12 µM of cabozantinib (Cbz) at four time-points were analyzed with an immunoblotting assay with specific antibodies for the indicated proteins. GAPDH expression was selected as the loading control of the samples. A stripping and re-probing protocol was performed to remove the antibodies from the membranes and re-probed with another antibody.

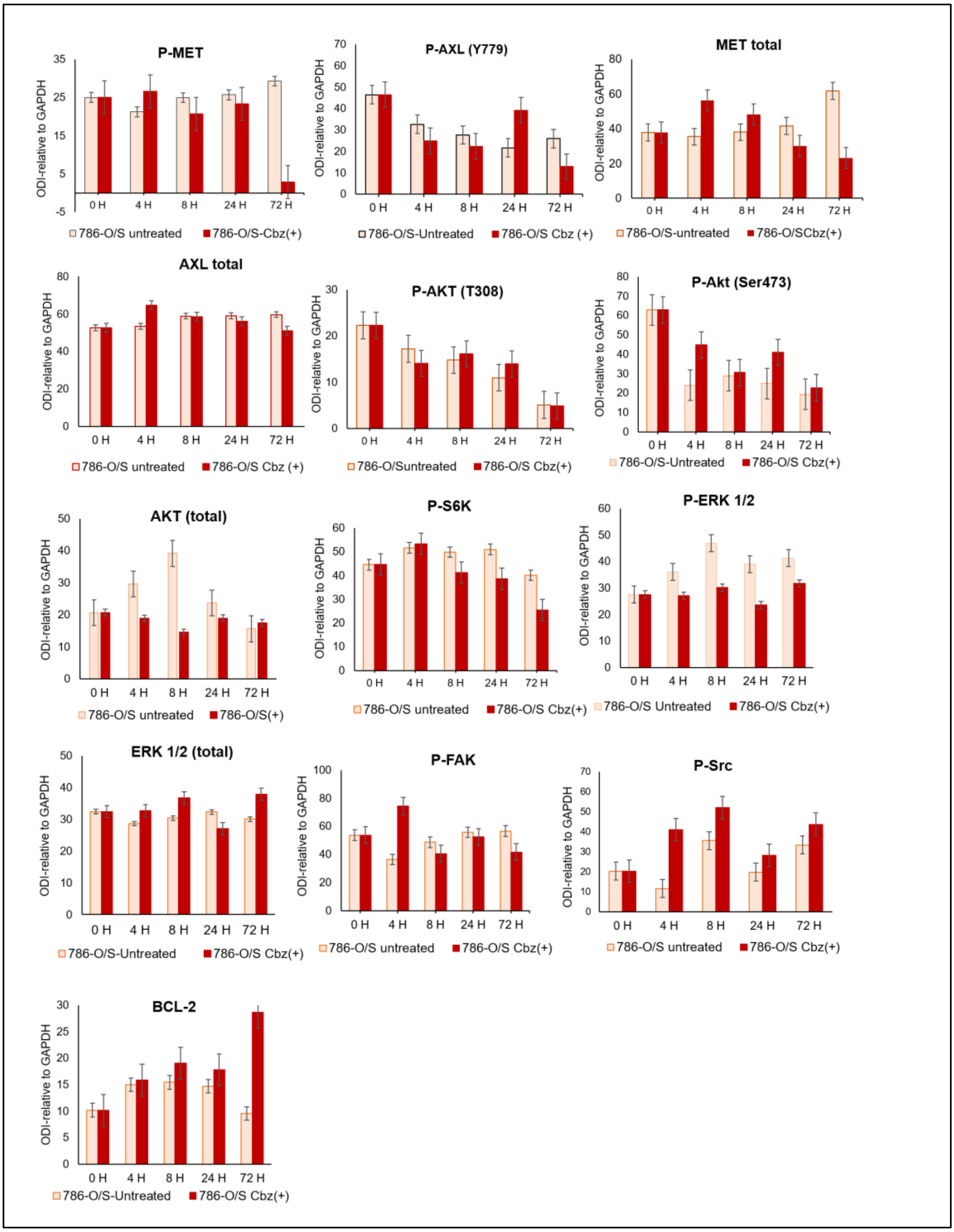

Figure S6. Quantification of Western blotting to analyze the effect of cabozantinib on the receptor tyrosine kinases MET, AXL and the downstream signal transduction following cabozantinib treatment in the sunitinib-resistant 786-O/S cell line. Densitometry analysis referring to Figures 2, 3 and Supplementary figure S5. The densitometry readings of the band's intensity were performed using *ImageJ* software [1]. The data evaluations, normalizing data representations, were performed using Microsoft Excel. Briefly, the pixel intensity of the target proteins and their backgrounds, alongside their loading control values, were inverted into binary values. Subsequently, the optical density (OD) of the target protein bands and loading controls were calculated by deducting the background to achieve high accuracy values. Finally, the ODI of the target protein was divided by the ODI of the loading control GAPDH.

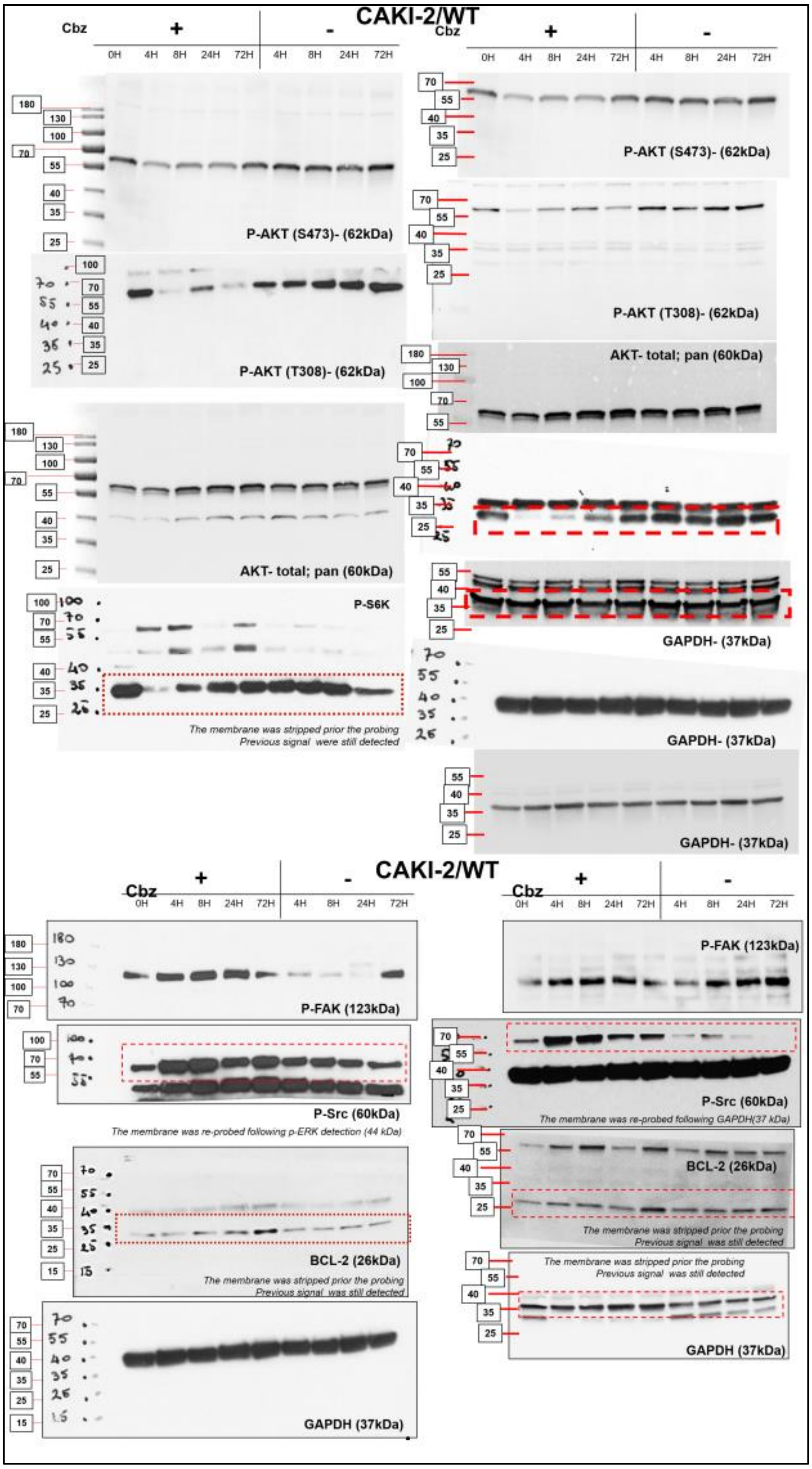

Figure S7. Effect of cabozantinib on signal transduction in treatment-naïve human RCC Caki-2/WT cell lines. Original immunoblotting results referring to Figure 4. Cell lysates obtained from wild-type Caki-2/WT cells treated (+) and untreated (-) with 12 μM of cabozantinib (Cbz) at four time-points were analyzed with an immunoblotting assay with specific antibodies for the indicated proteins. The GAPDH expression was selected as the loading control of the samples. A stripping and re-probing protocol was performed to remove the antibodies from the membranes and re-probed with another antibody.

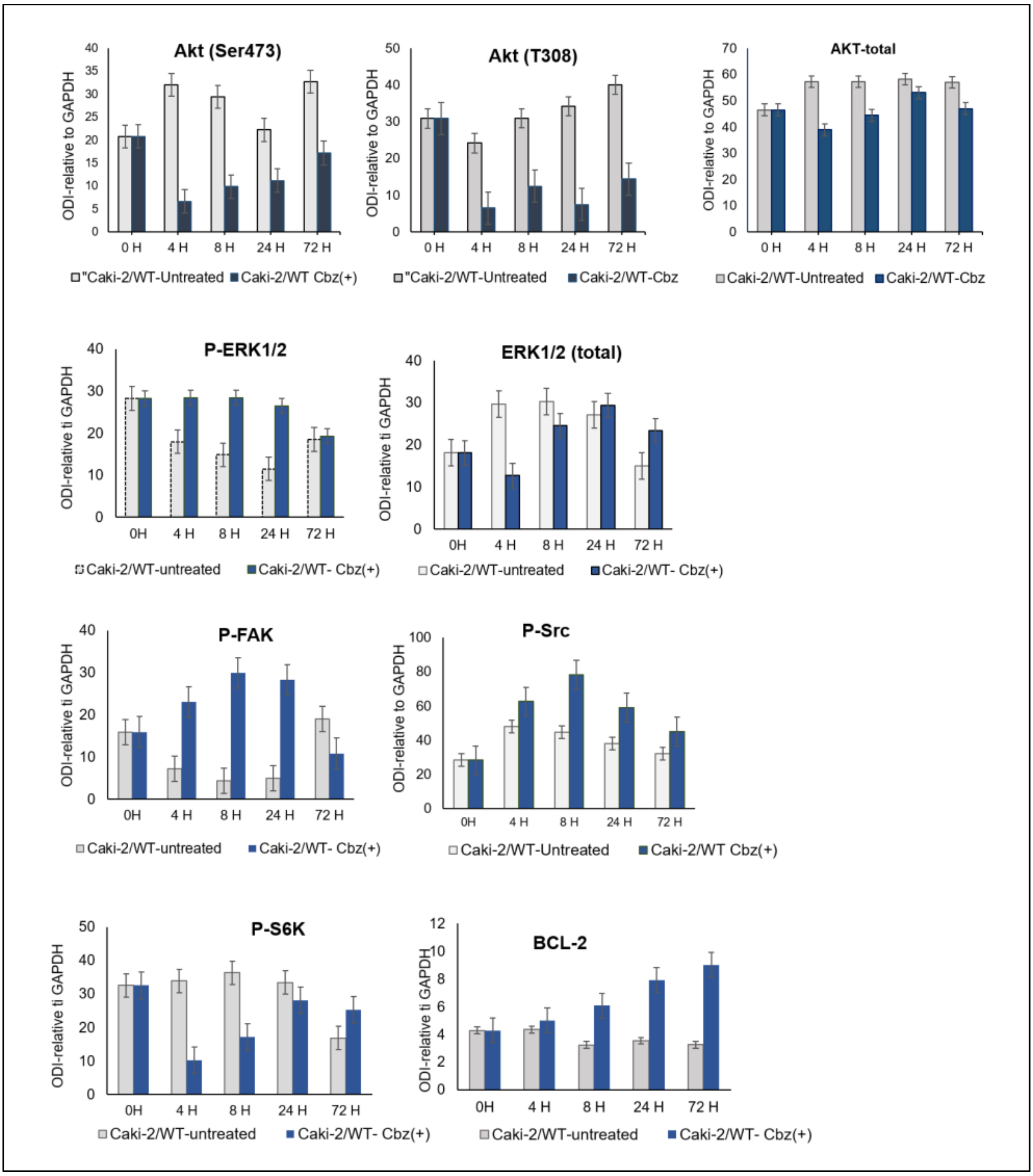

Figure S8. Quantification of Western blotting to analyze the effect of cabozantinib on signal transduction in treatment-naïve human RCC Caki-2/WT cell line. Densitometry analysis referring to Figure 5 and Supplementary figure S7. The densitometry readings of the band's intensity were performed using *ImageJ* software [1]. The data evaluations, normalizing data representations, were performed using Microsoft Excel. Briefly, the pixel intensity of the target proteins and their backgrounds, alongside their loading control values, were inverted into binary values. Subsequently, the optical density (OD) of the target protein bands and loading controls were calculated by deducting the background to achieve high accuracy values. Finally, the ODI of the target protein was divided by the ODI of the loading control GAPDH.

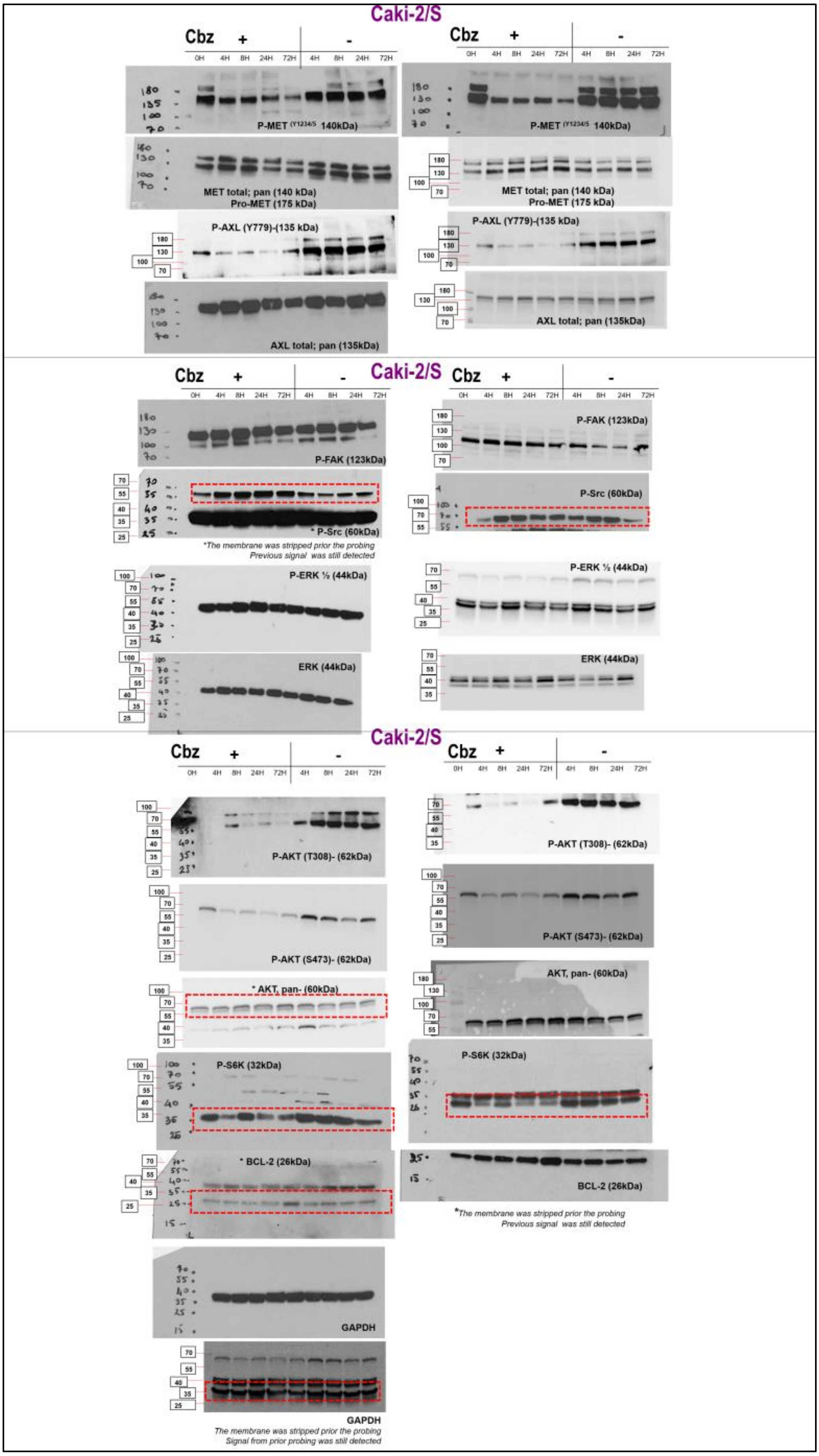

Figure S9. Effect of cabozantinib on receptor tyrosine kinases MET, AXL and the downstream signal transduction in the sunitinib-resistant Caki-2/S cell line. Original immunoblotting results referring to Figures 4 and 5. Cell lysates obtained from Caki-2/S cells that were either treated (+) or untreated (-) with 12 μM of cabozantinib (Cbz) at four time-points were analyzed with an immunoblotting assay with specific antibodies for the indicated proteins. GAPDH expression was selected as the loading control of the samples. A stripping and re-probing protocol was performed to remove the antibodies from the membranes and re-probed with another antibody.

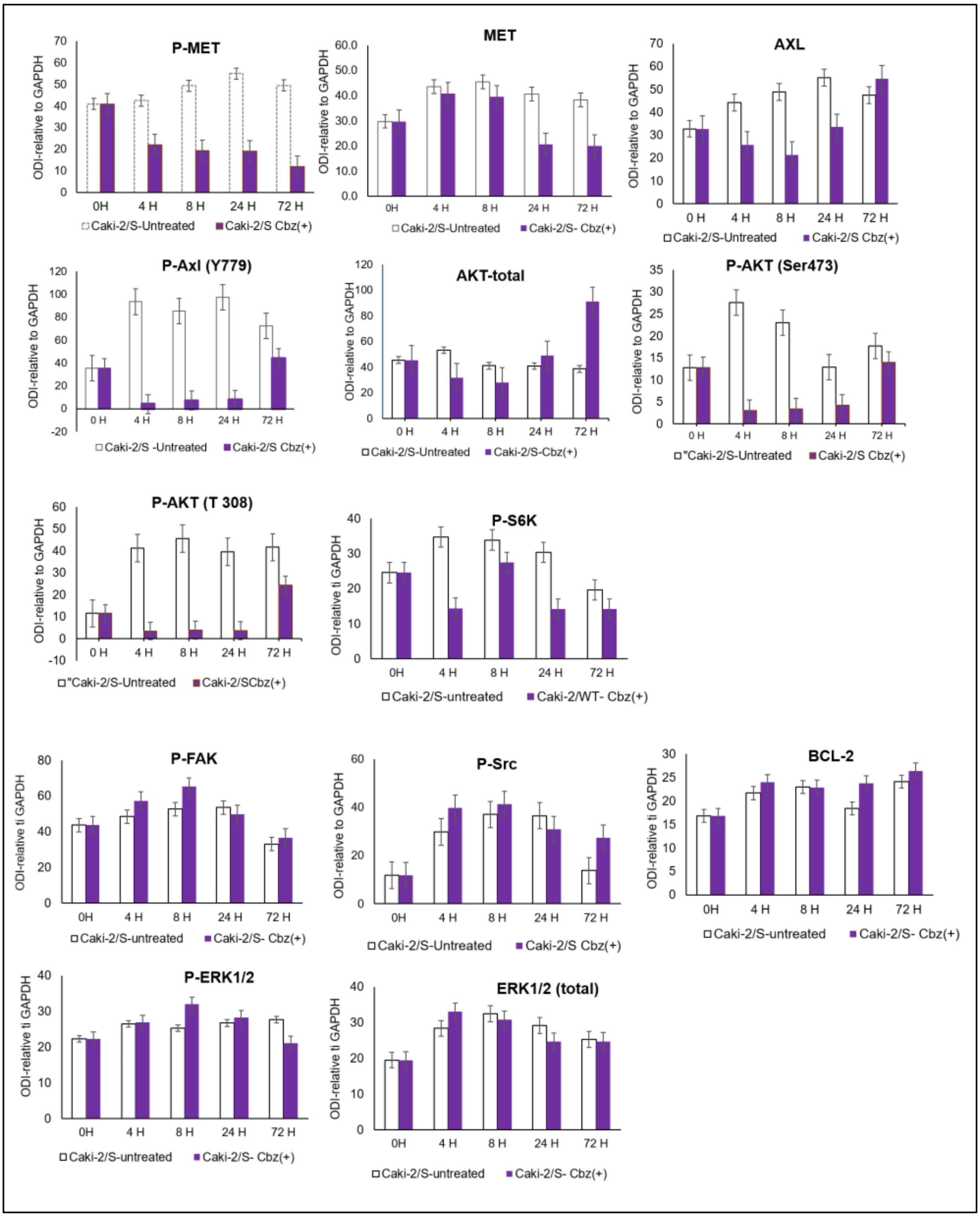

Figure S10. Quantification of Western blotting to analyze the effect of cabozantinib on the receptor tyrosine kinases MET, AXL and the downstream signal transduction following cabozantinib treatment in the sunitinib-resistant Caki-2/S cell line. Densitometry analysis referring to Figures 4, 5 and Supplementary figure S10. The densitometry readings of the band's intensity were performed using *ImageJ* software [1]. The data evaluations, normalizing data representations, were performed using Microsoft Excel. Briefly, the pixel intensity of the target proteins and their backgrounds, alongside their loading controls, were inverted into binary values. Subsequently, the optical density (OD) of the target protein bands and loading controls were calculated by deducting the background to achieve high accuracy values. Finally, the ODI of the target protein was divided by the ODI of the loading control GAPDH.

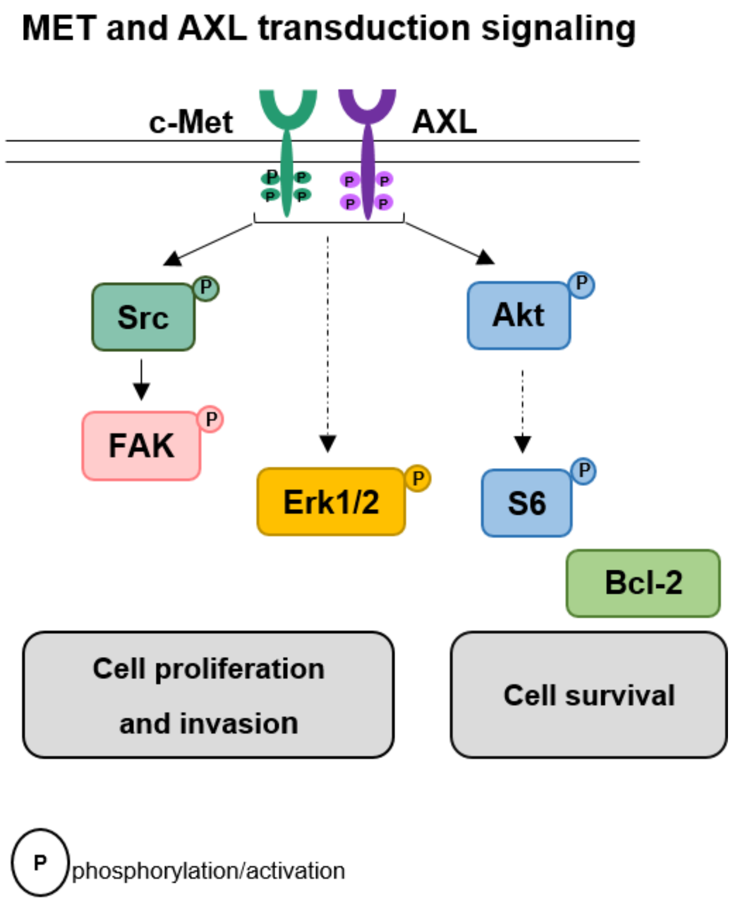

Figure S11. Illustration of the receptor tyrosine kinases MET and AXL-mediated signaling. Activation of receptors MET and AXL (via phosphorylation) was described to act as an alternative pathway contributing to treatment resistance to sunitinib during the first line therapy in RCC. Dysregulation of MET and AXL correlated with aggressive tumor behavior. Cabozantinib, as a multi-kinase inhibitor, cabozantinib targets MET and AXL signaling important to tumor cell proliferation, invasion and metastasis.
